# Supplementary material for: Trichilones A–E: New Limonoids from Trichilia adolfi
Source: Molecules. 2021 May 21;26(11):3070. doi: 10.3390/molecules26113070 (PMC8196563; doi:10.3390/molecules26113070)

Figure S1: ^{1}H NMR of compound 1 ($CDCl_3$)

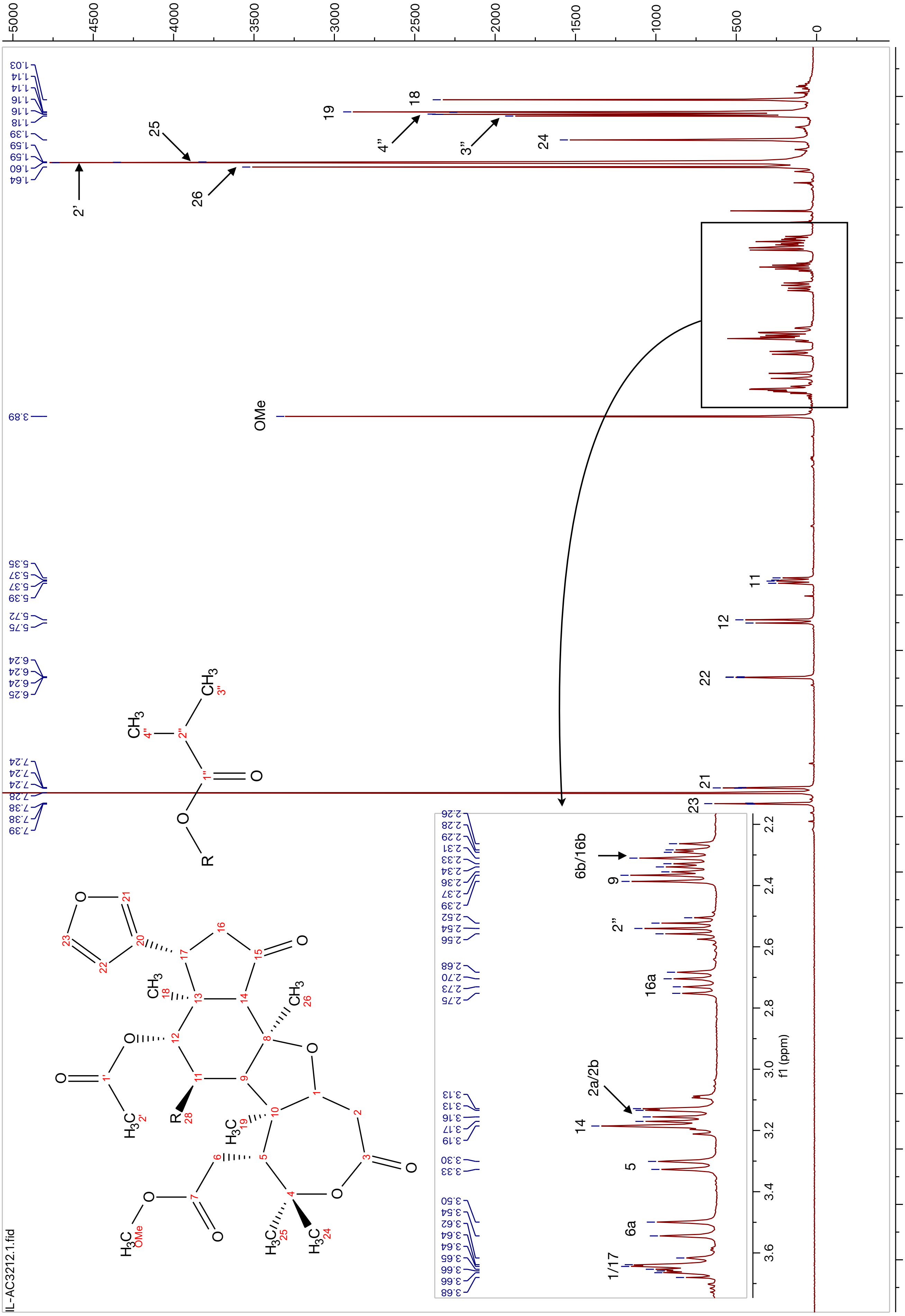

Figure S2: ^{13}C NMR of compound 1 ($CDCl_3$)

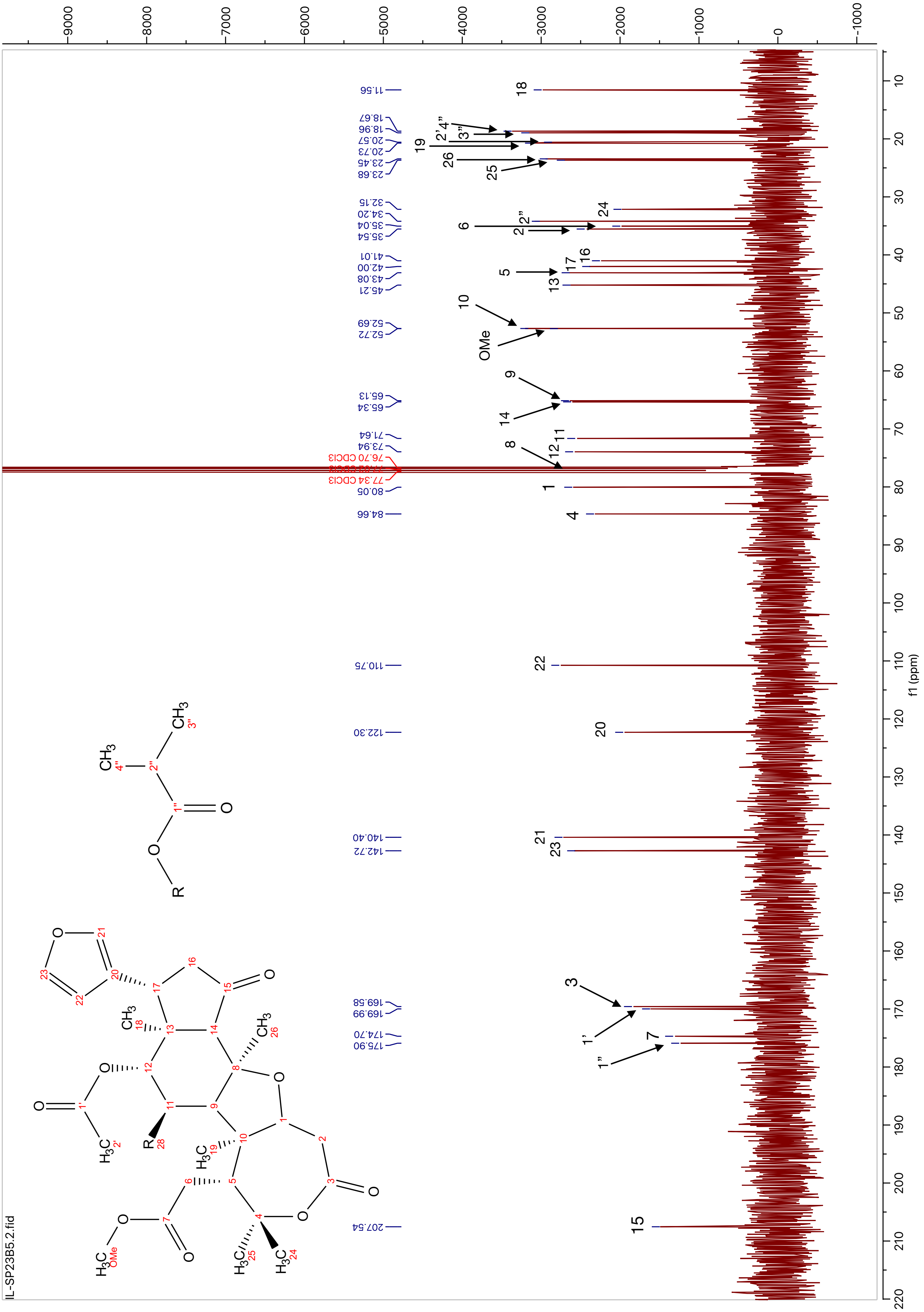

Figure S3: COSY experiment of compound 1 ($CDCl_3$)

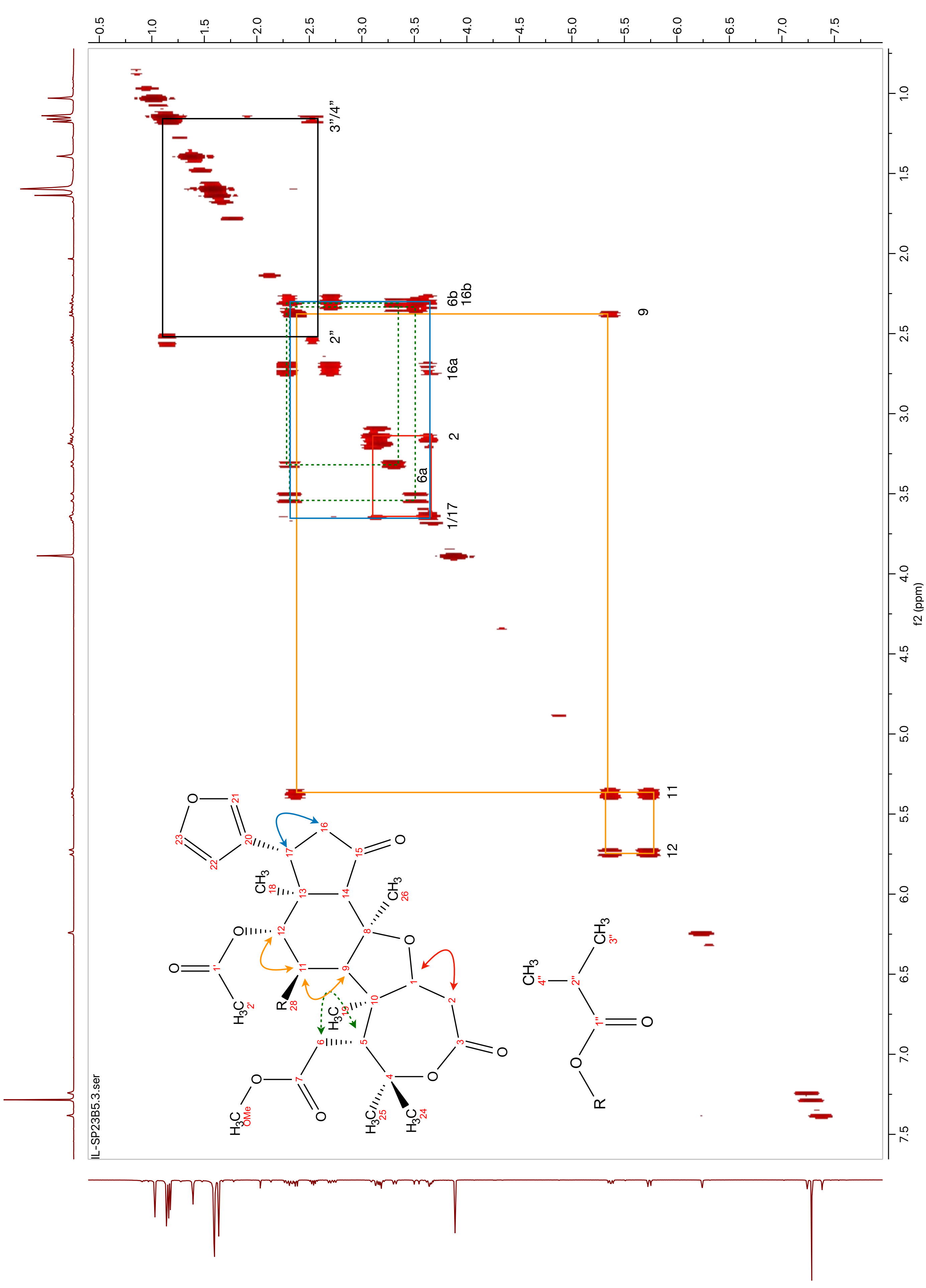

Figure S4: HMQC experiment of compound 1 (CDCl3)$_3$)

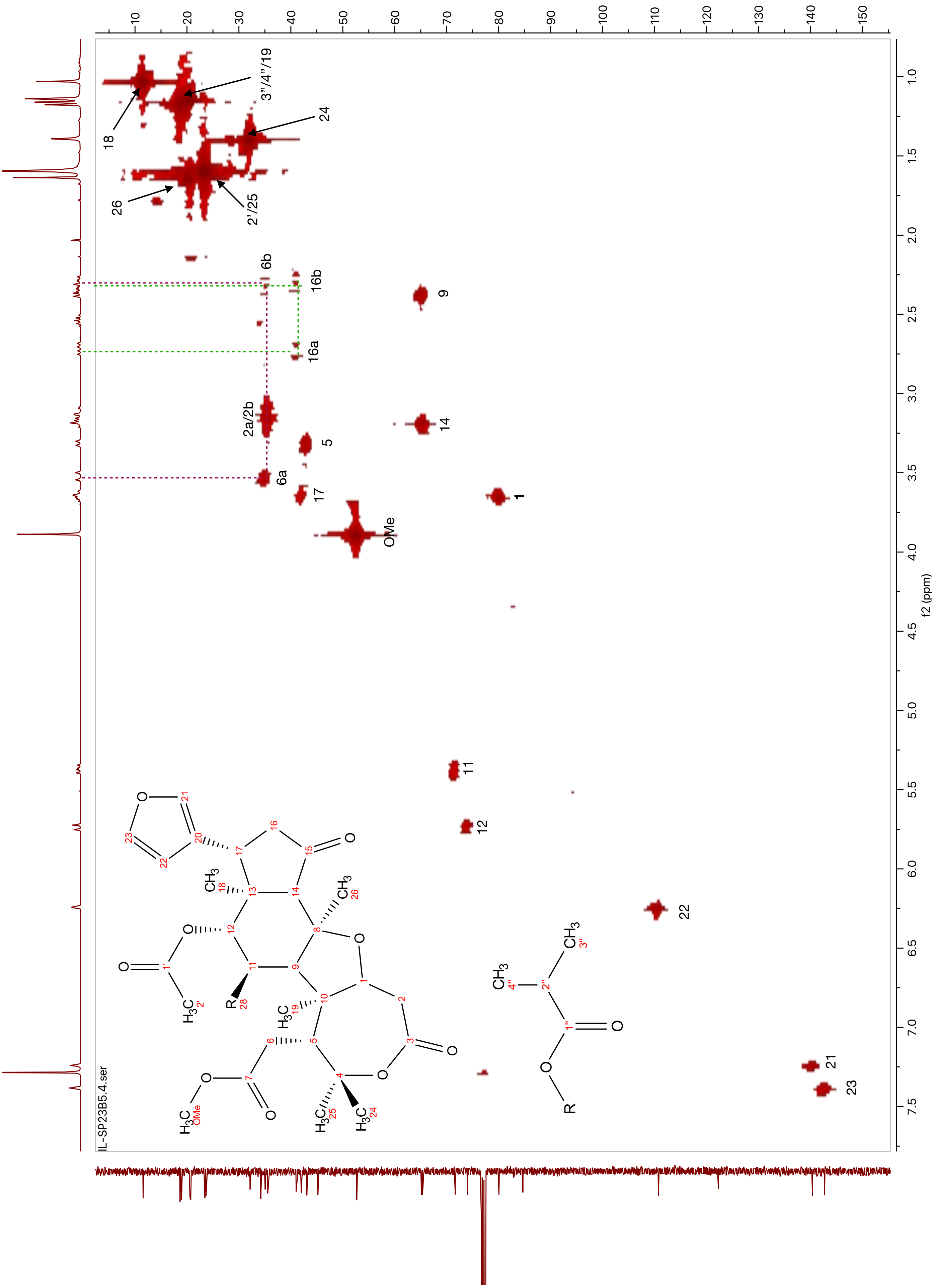

Figure S5: HMBC experiment of compound 1 ($CDCl_3$)

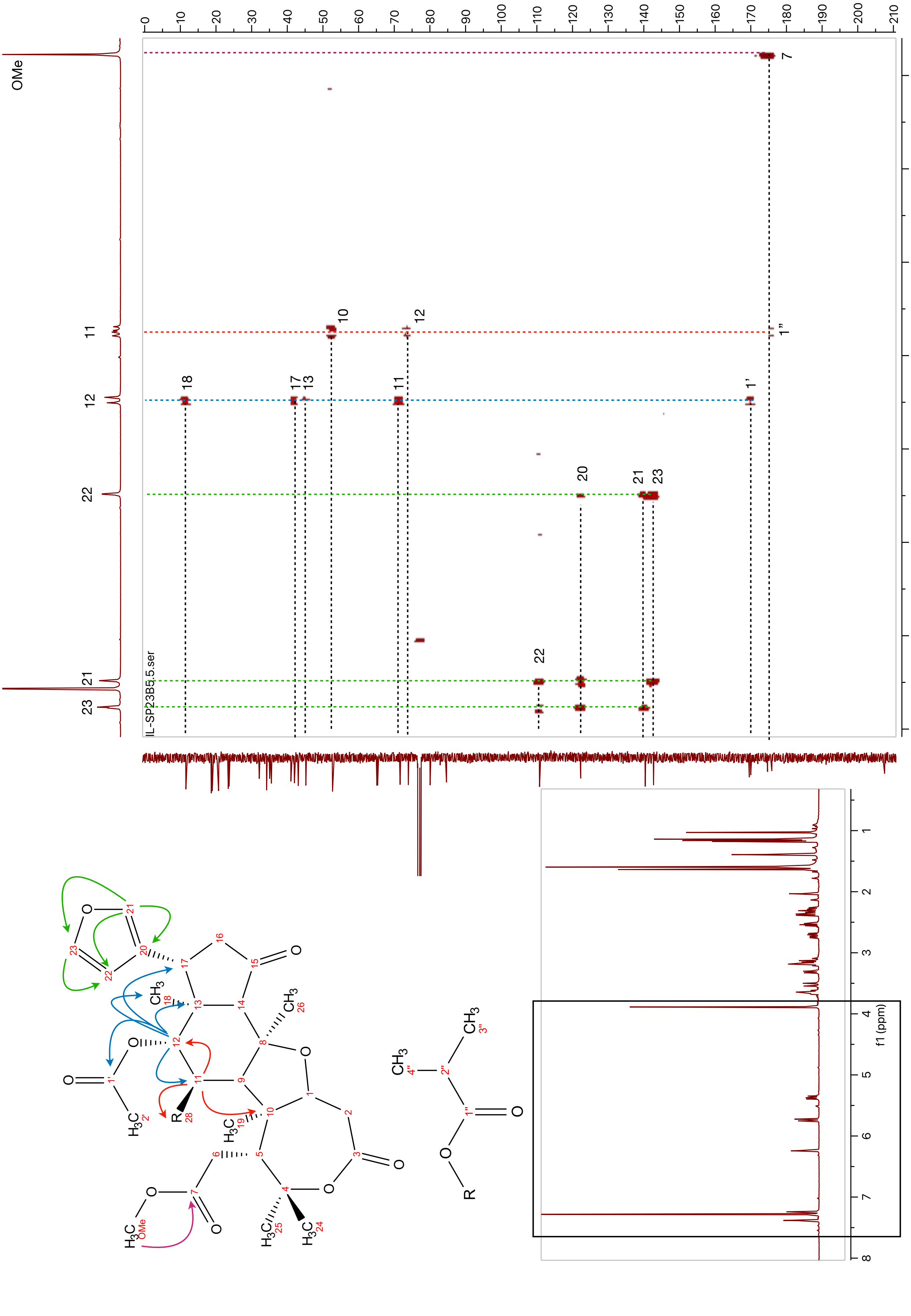

Figure S6: HMBC experiment of compound 1 ($CDCl_3$)

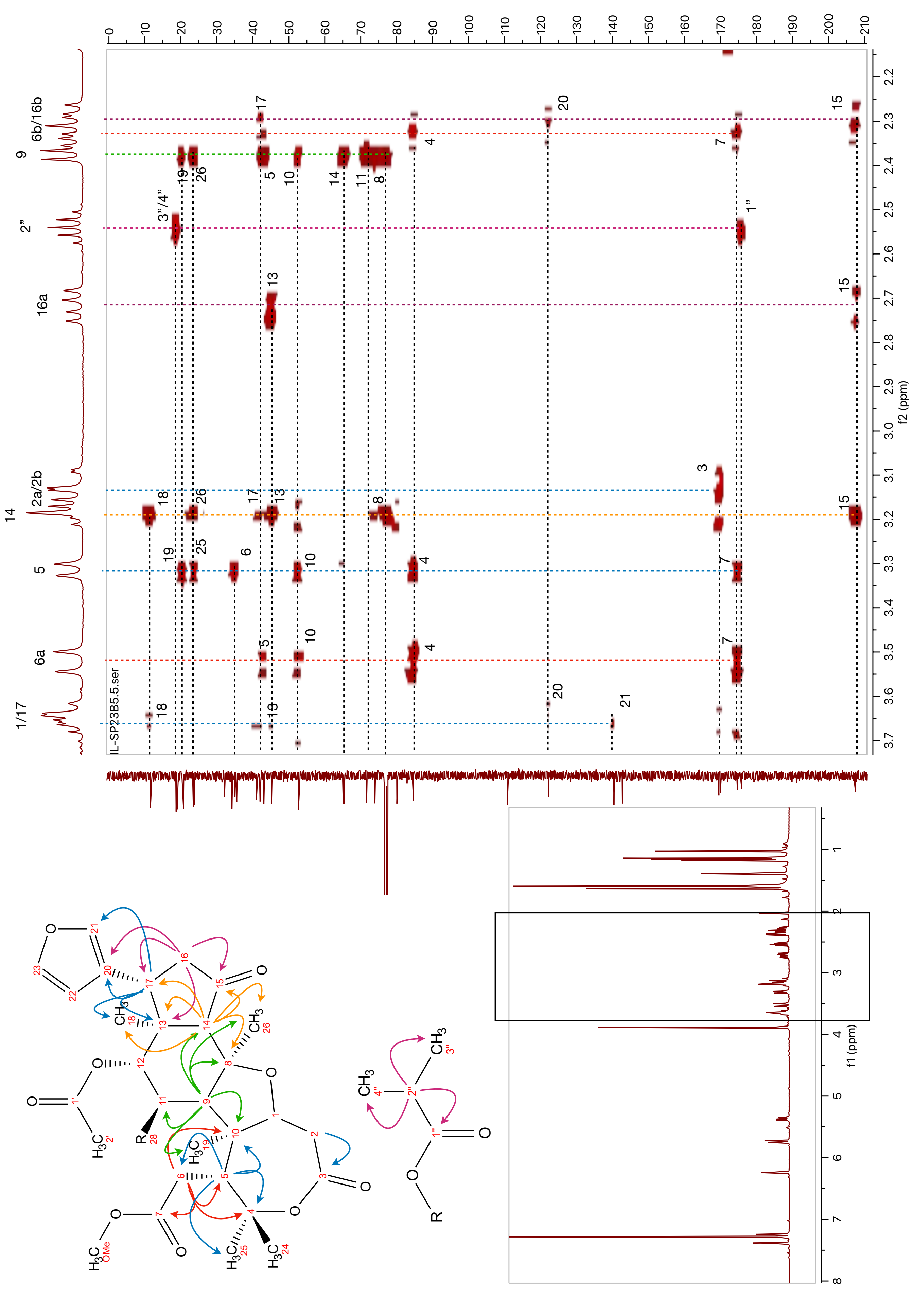

Figure S7: HMBC experiment of compound 1 ($CDCl_3$)

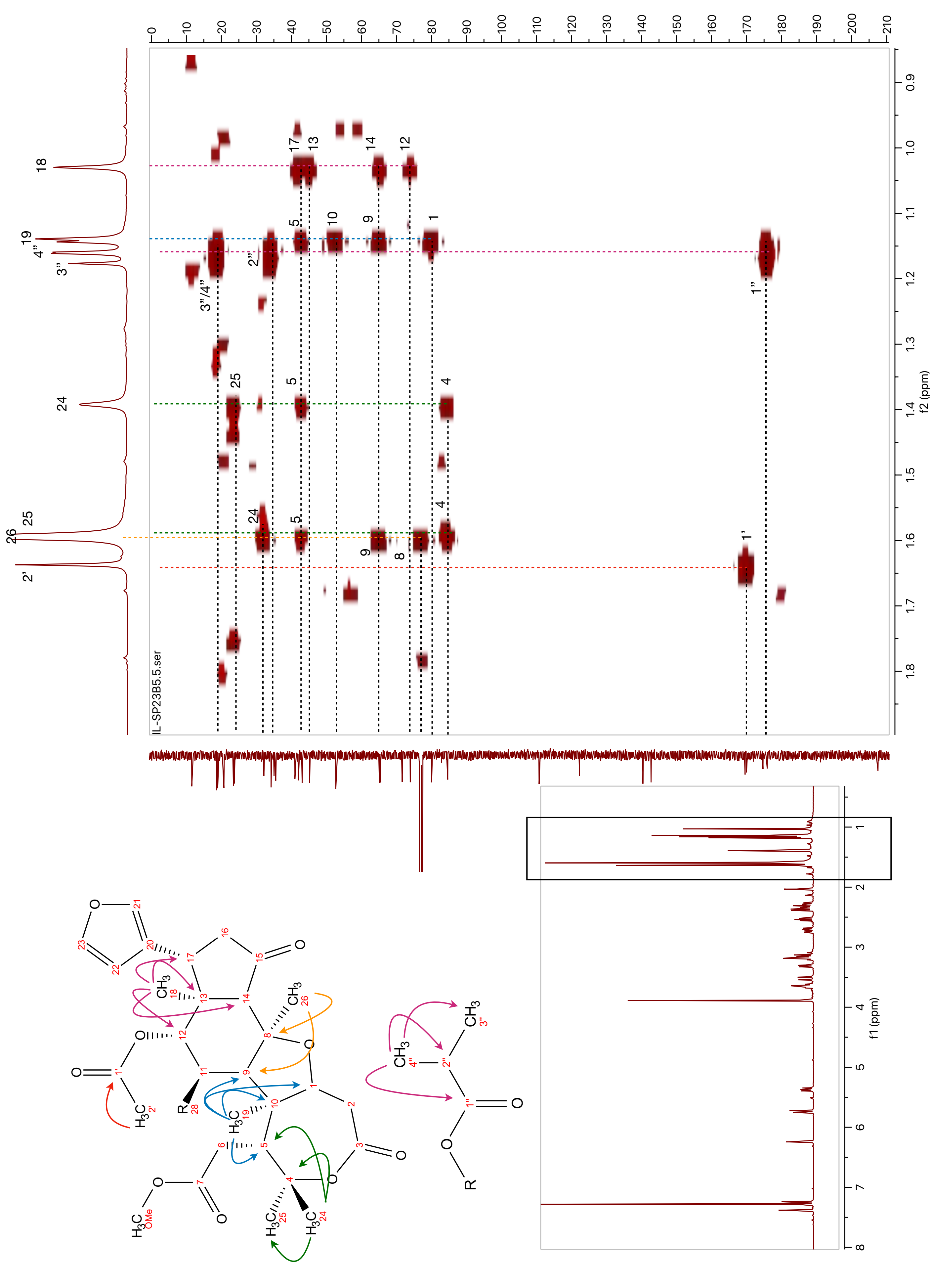

Figure S8: NOESY experiment of compound 1 ($CDCl_3$)

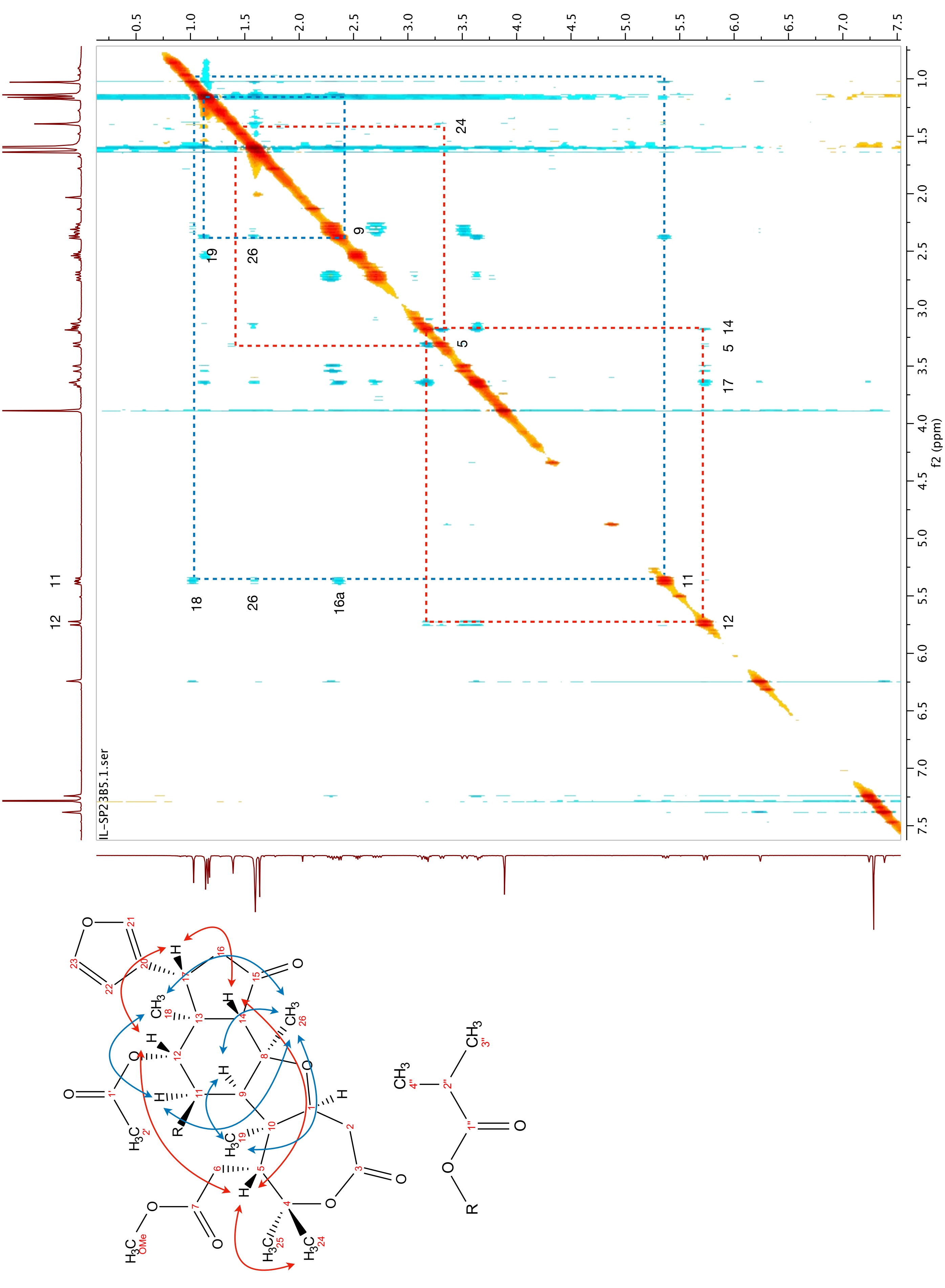

Figure S9: HRMS and IR spectra of compound 1

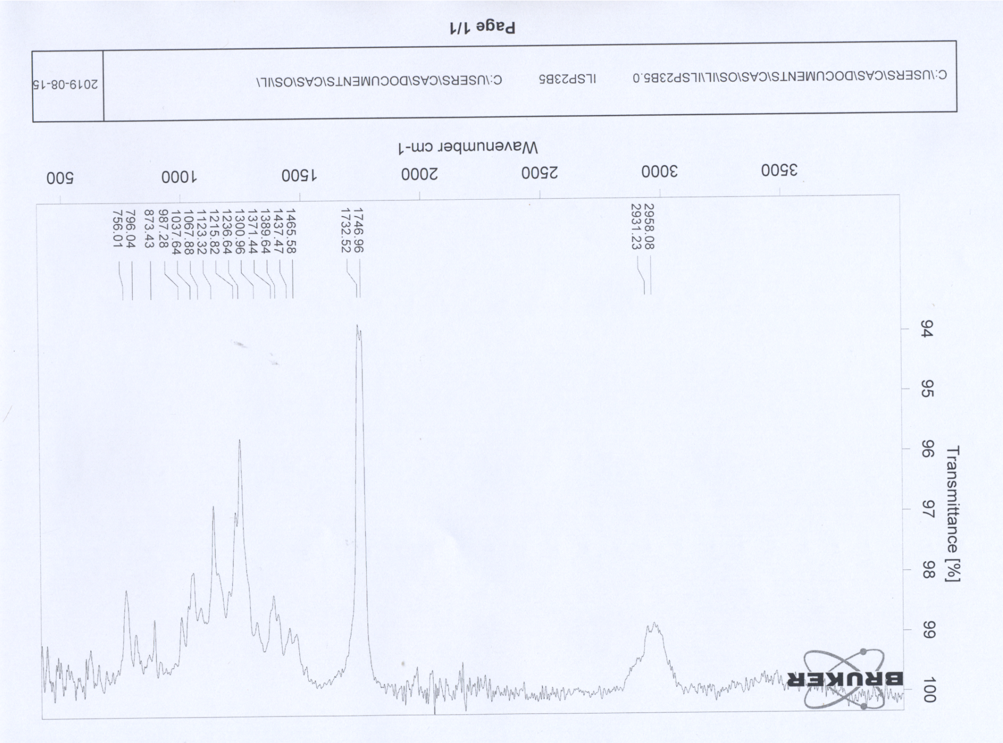

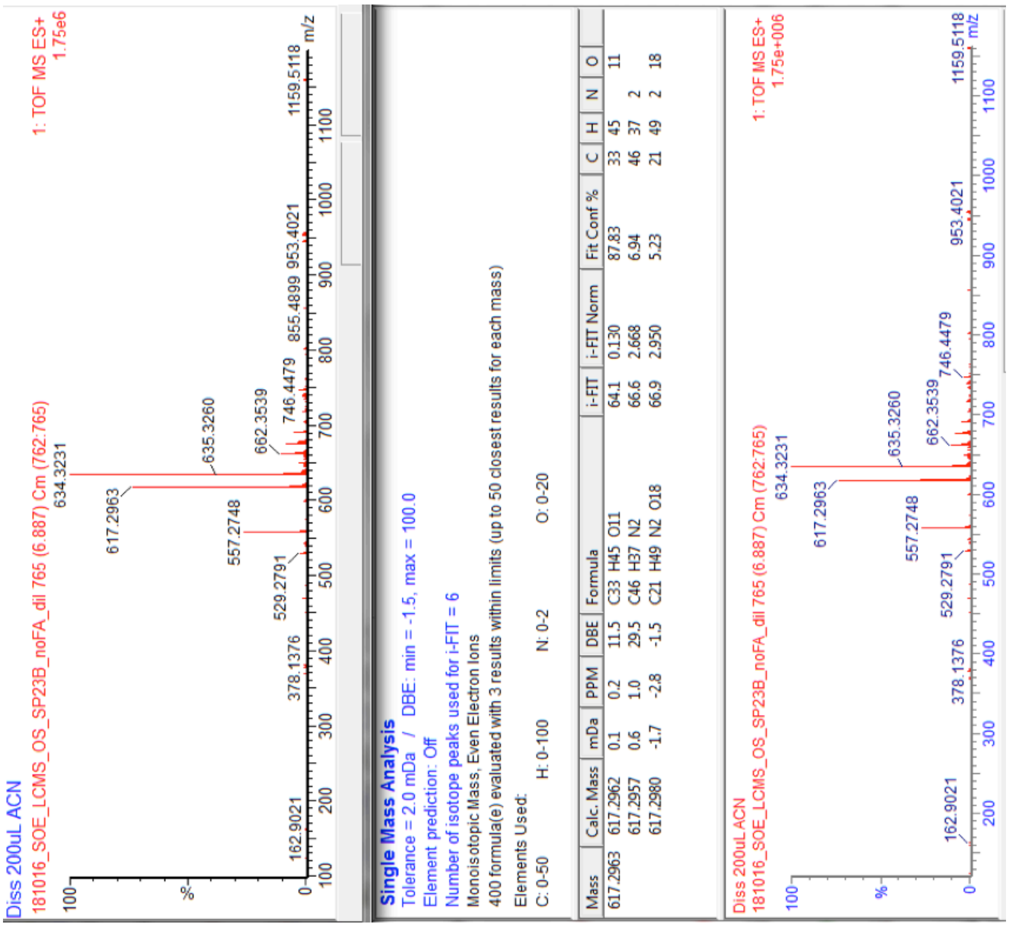

Figure S10: ^1H NMR of compound 2 ($CDCl_3$)

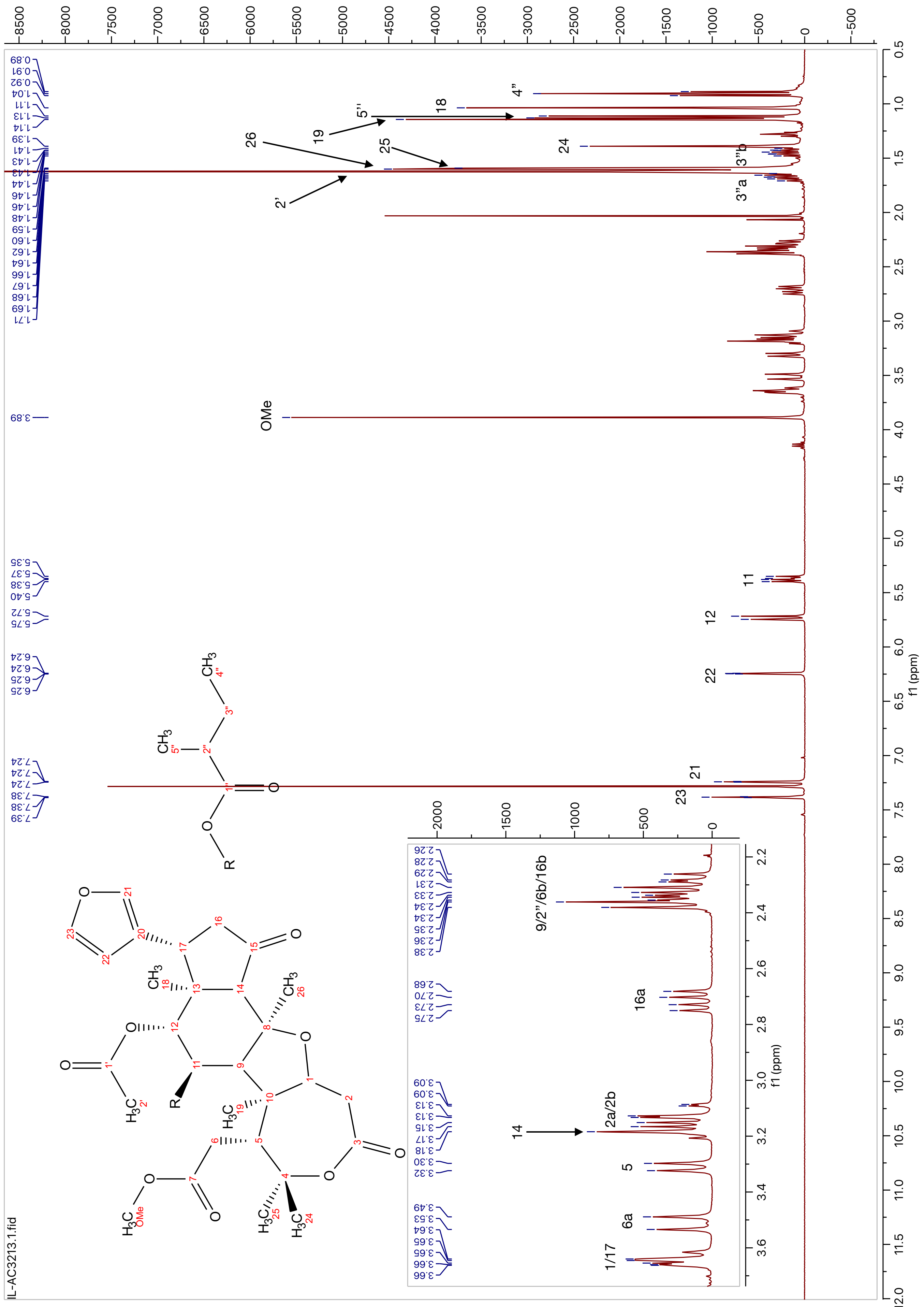

Figure S11: ^{13}C NMR of compound 2 ($CDCl_3$)

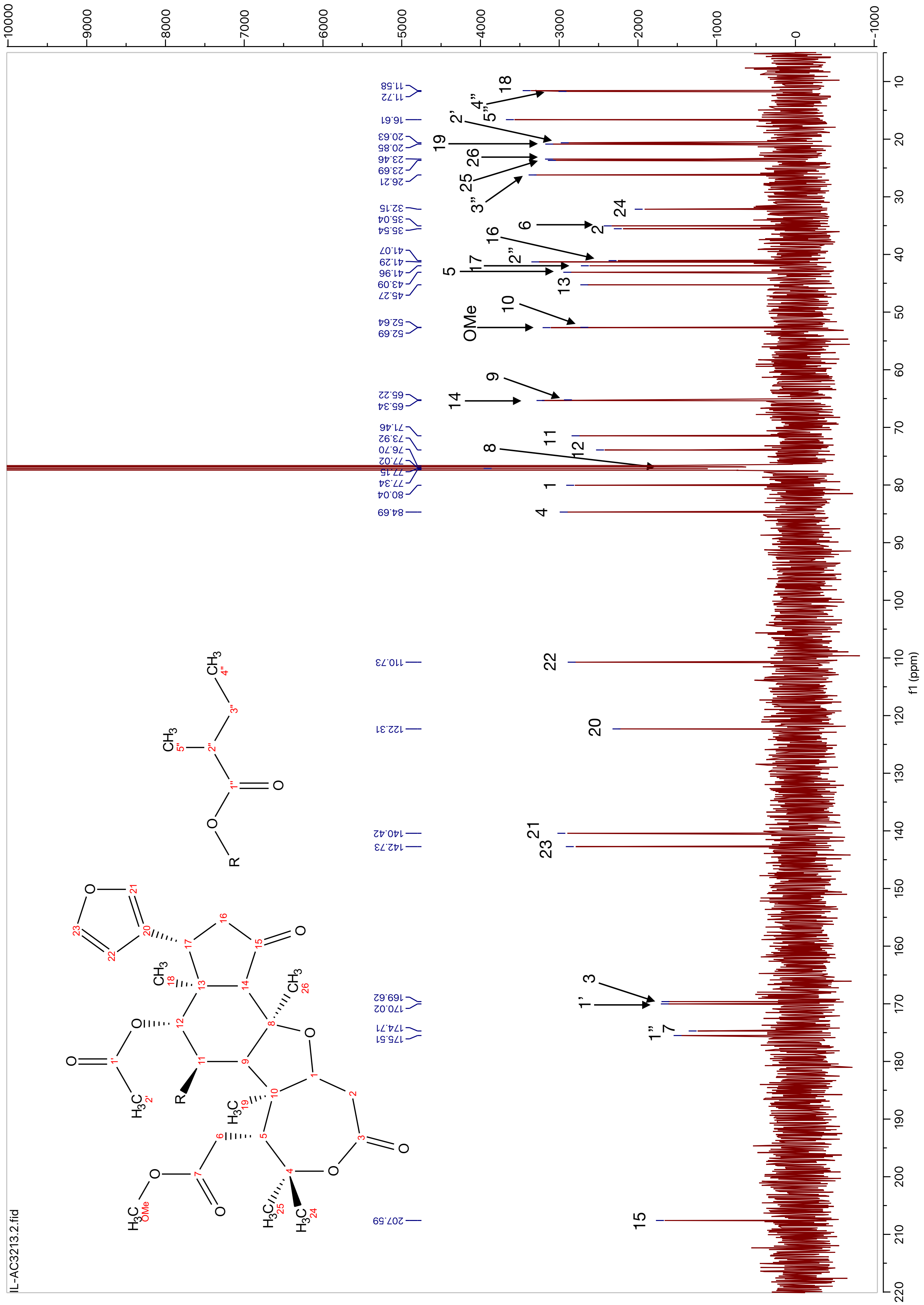

Figure S12: ^{13}C NMR and DEPT135 of compound 2 ($CDCl_3$)

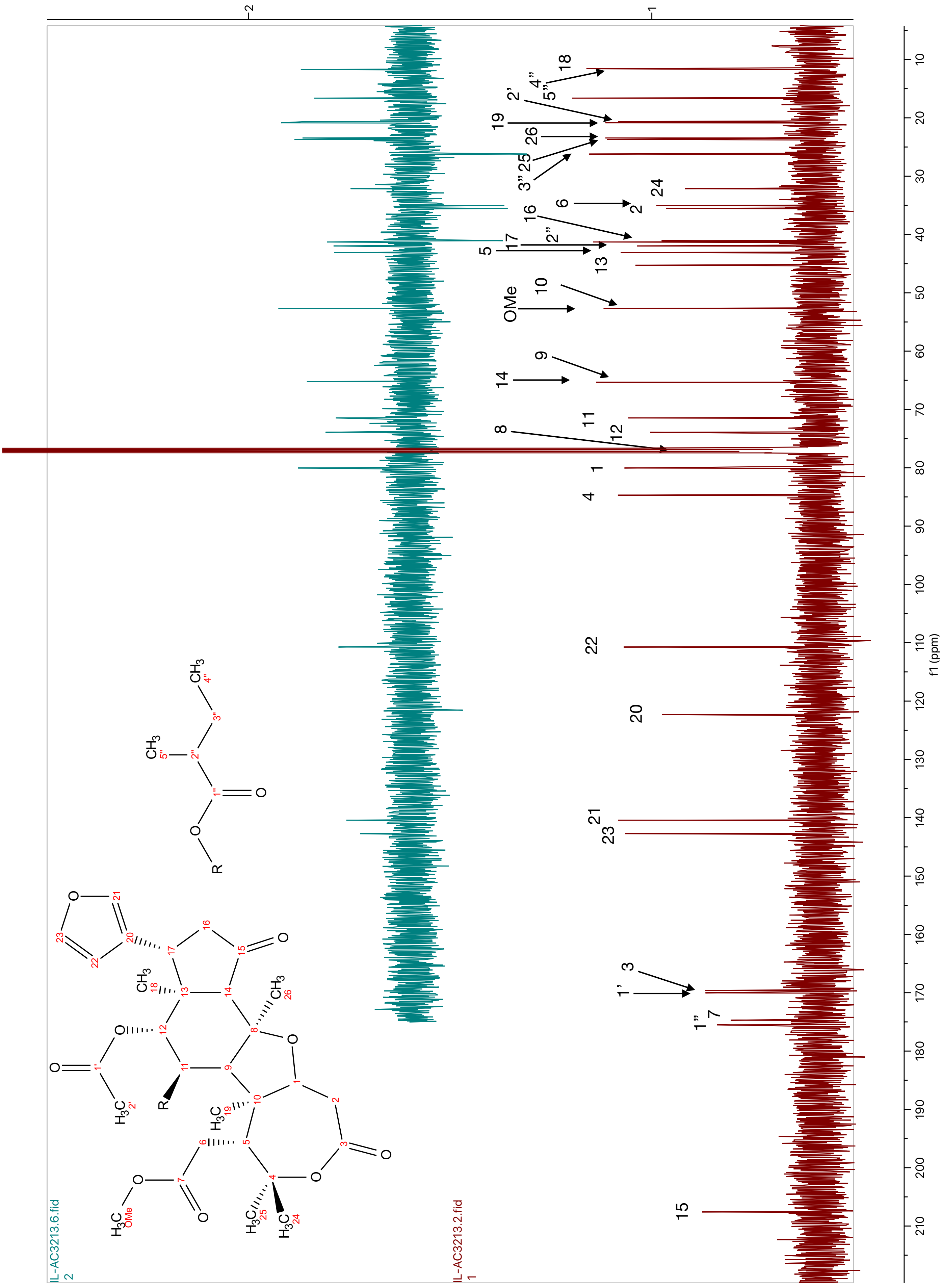

Figure S13: COSY experiment of compound 2 ($CDCl_3$)

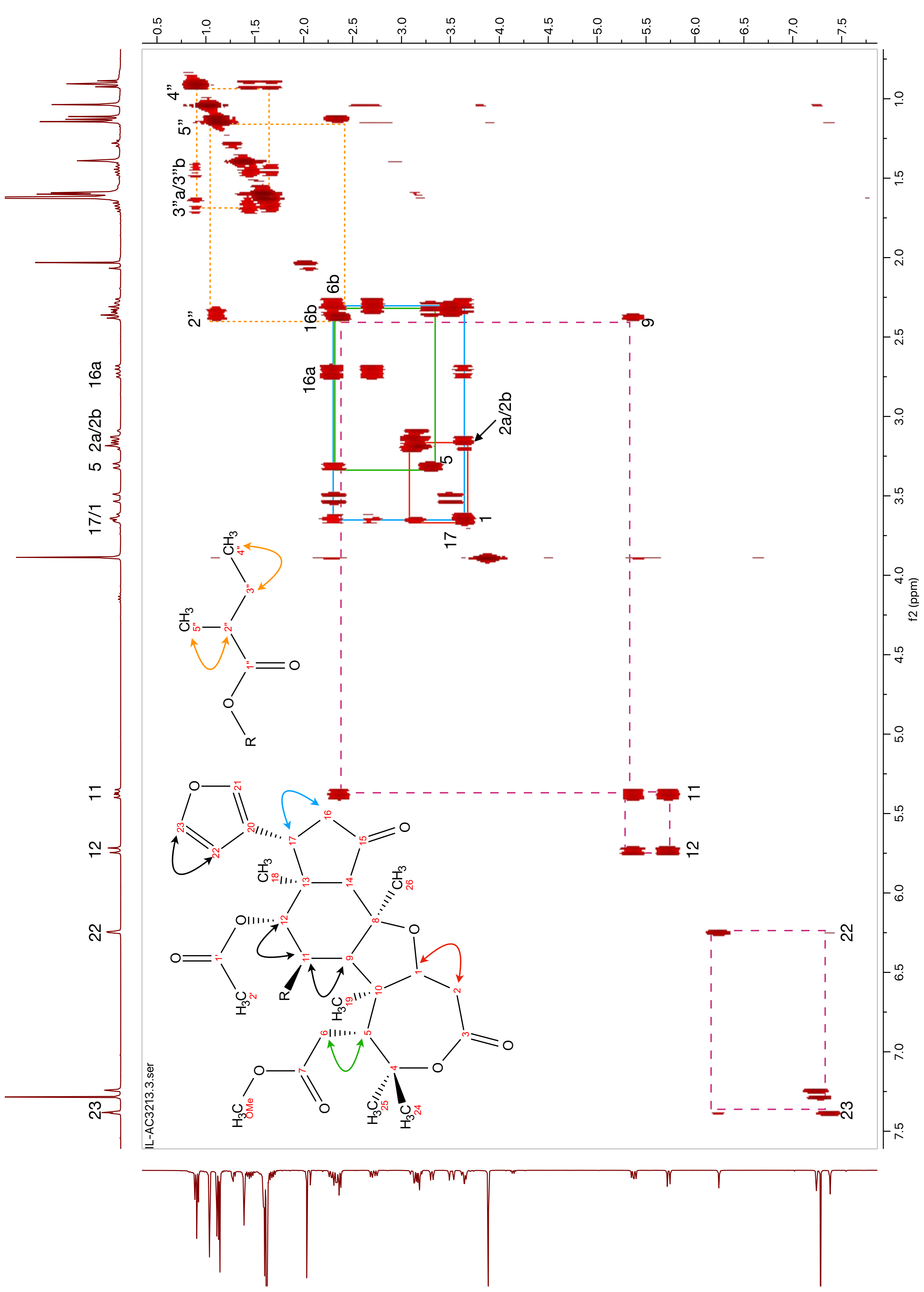

Figure S14: HMQC experiment of compound 2 (CDCl3)

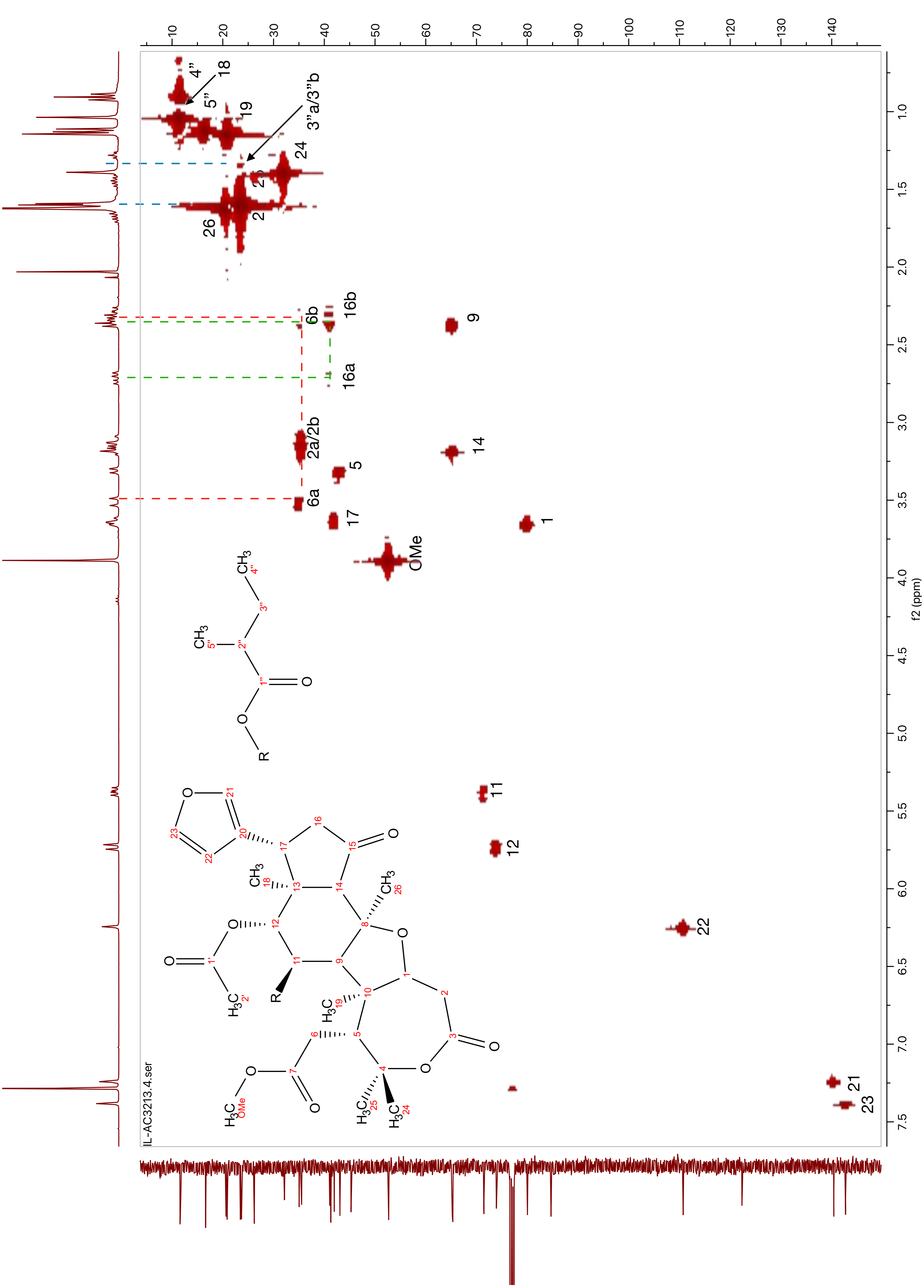

Figure S15: HMBC experiment of compound 2 ($CDCl_3$)

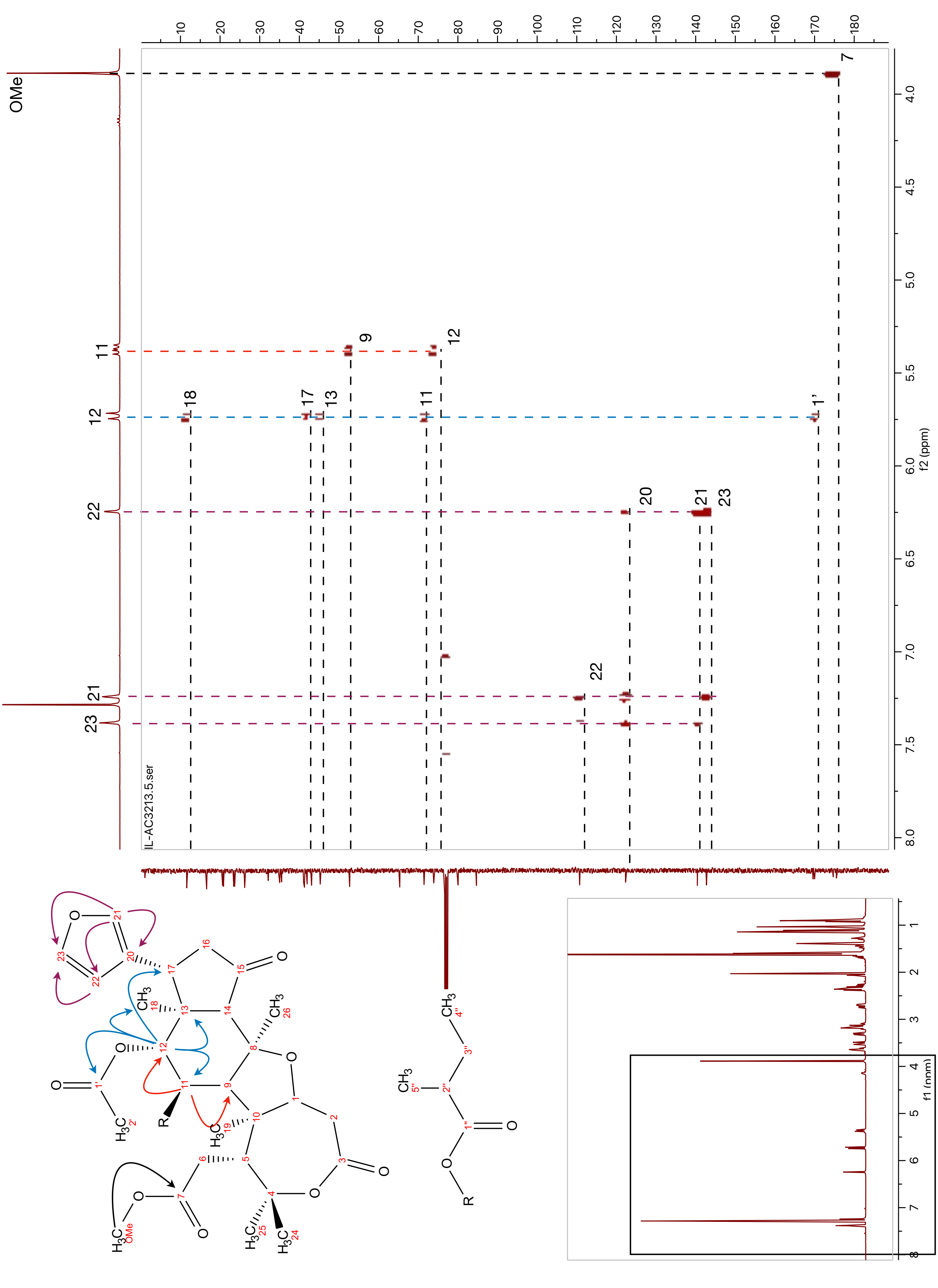

Figure S16: HMBC experiment of compound 2 ($CDCl_3$)

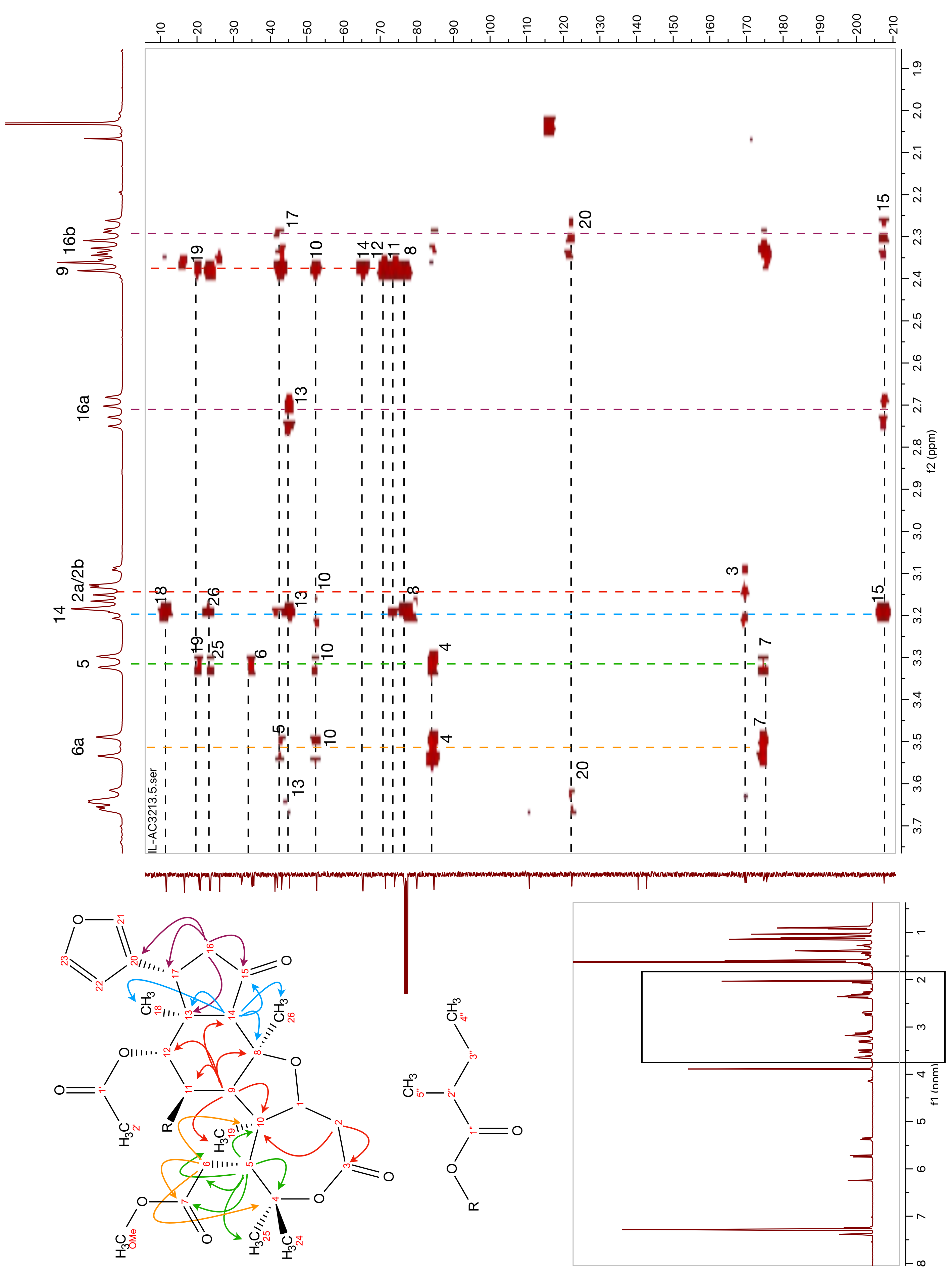

Figure S17: HMBC experiment of compound 2 ($CDCl_3$)

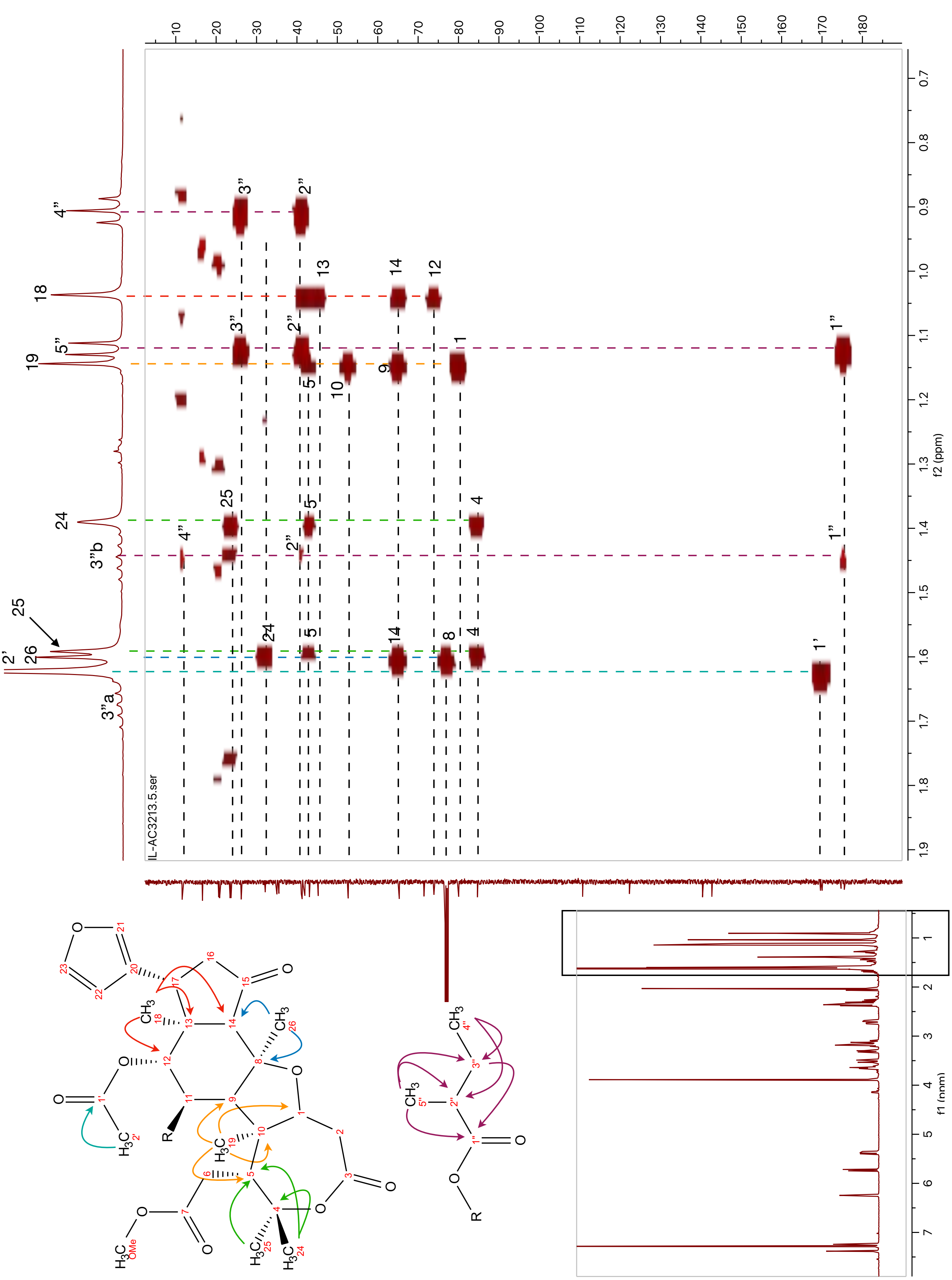

Figure S18: NOESY experiment of compound 2 ($CDCl_3$)

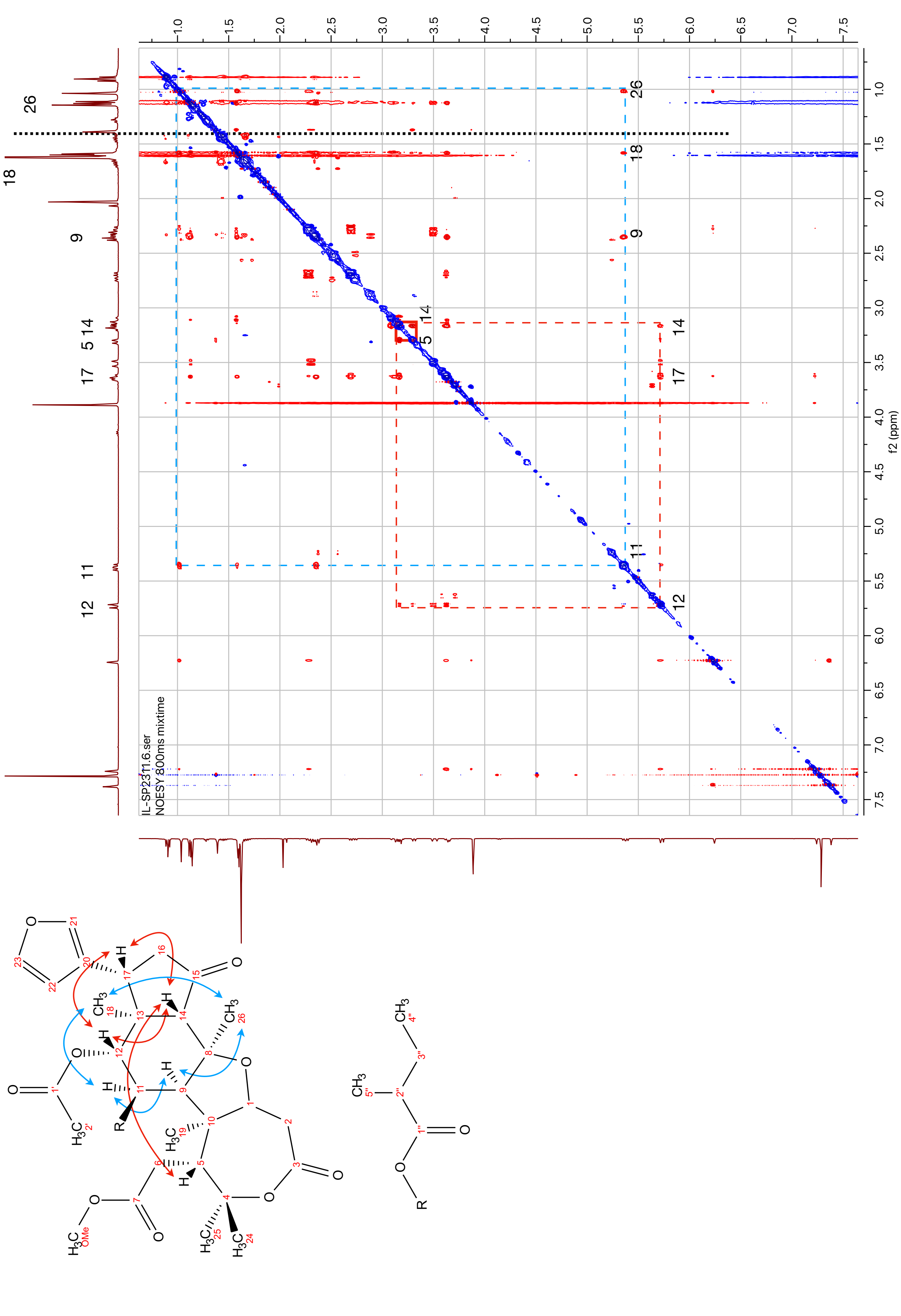

Figure S19: HRMS and IR spectra of compound 2

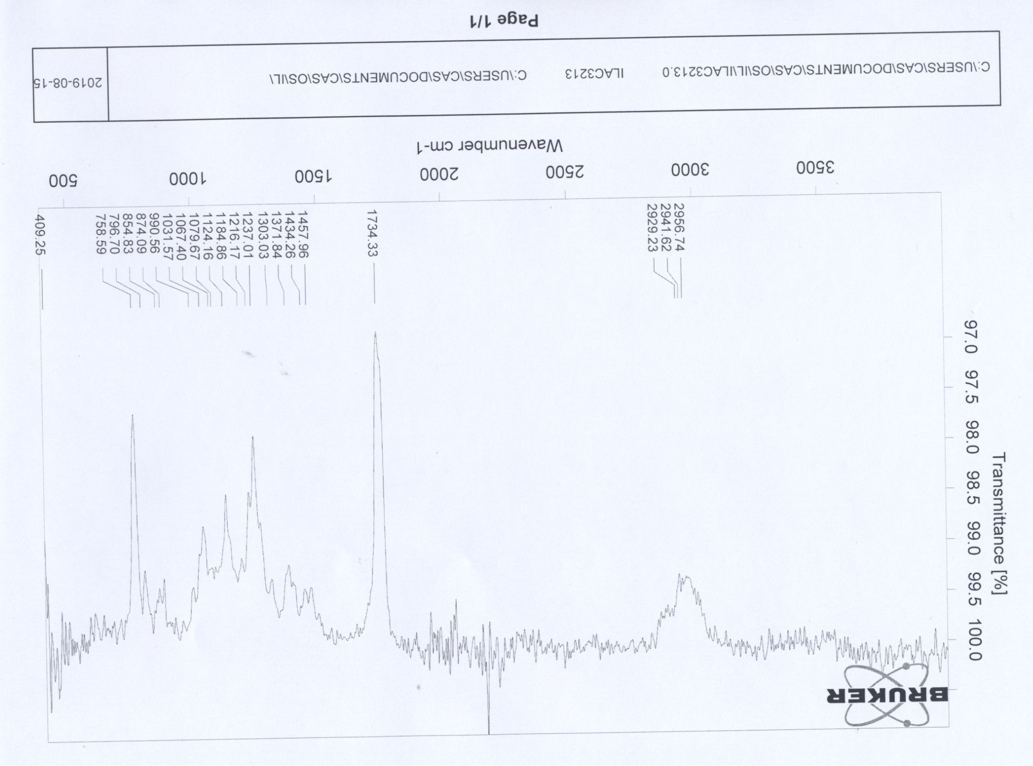

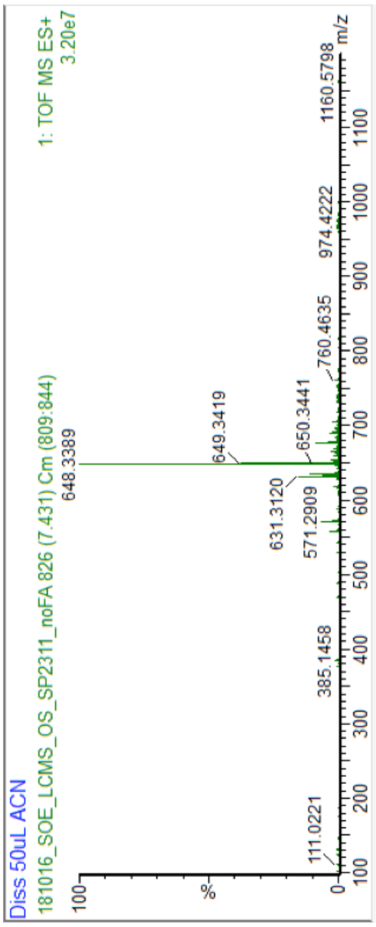

Single Mass Analysis
Tolerance = 2.0 mDa / DBE: min = -1.5, max = 100.0
Element prediction: Off
Number of isotope peaks used for i-FIT = 6

Monoisotopic Mass, Even Electron Ions
814 formula(e) evaluated with 4 results within limits (up to 50 closest results for each mass)
Elements Used:
C: 0-50 H: 0-100 N: 0-2 O: 0-20 Si: 0-1

Mass	Calc. Mass	mDa	PPM	DBE	Formula	i-FIT	i-FIT Norm	Fit Conf %	C	H	N	O	Si
631.3120	631.3118	0.2	0.3	11.5	C34 H47 O11	74.5	3.531	2.93	34	47		11	
	631.3113	0.7	1.1	29.5	C47 H39 N2	71.0	0.033	96.76	47	39	2		
	631.3110	1.0	1.6	2.5	C25 H51 N2 O14 Si	81.9	10.977	0.00	25	51	2	14	1
	631.3137	-1.7	-2.7	-1.5	C22 H51 N2 O18	76.7	5.761	0.31	22	51	2	18	

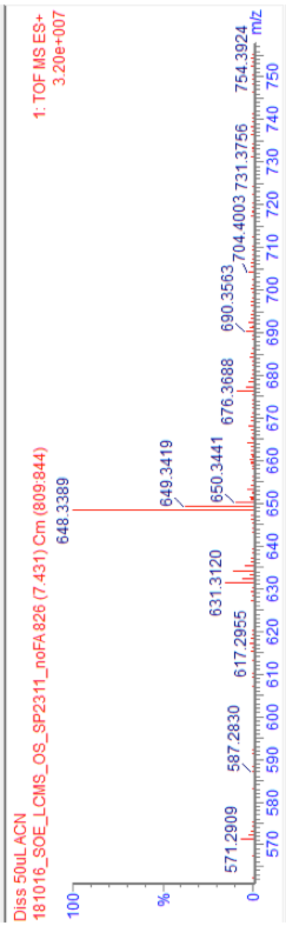

Figure S20: ^{1}H NMR of compound 3 ($CDCl_3$)

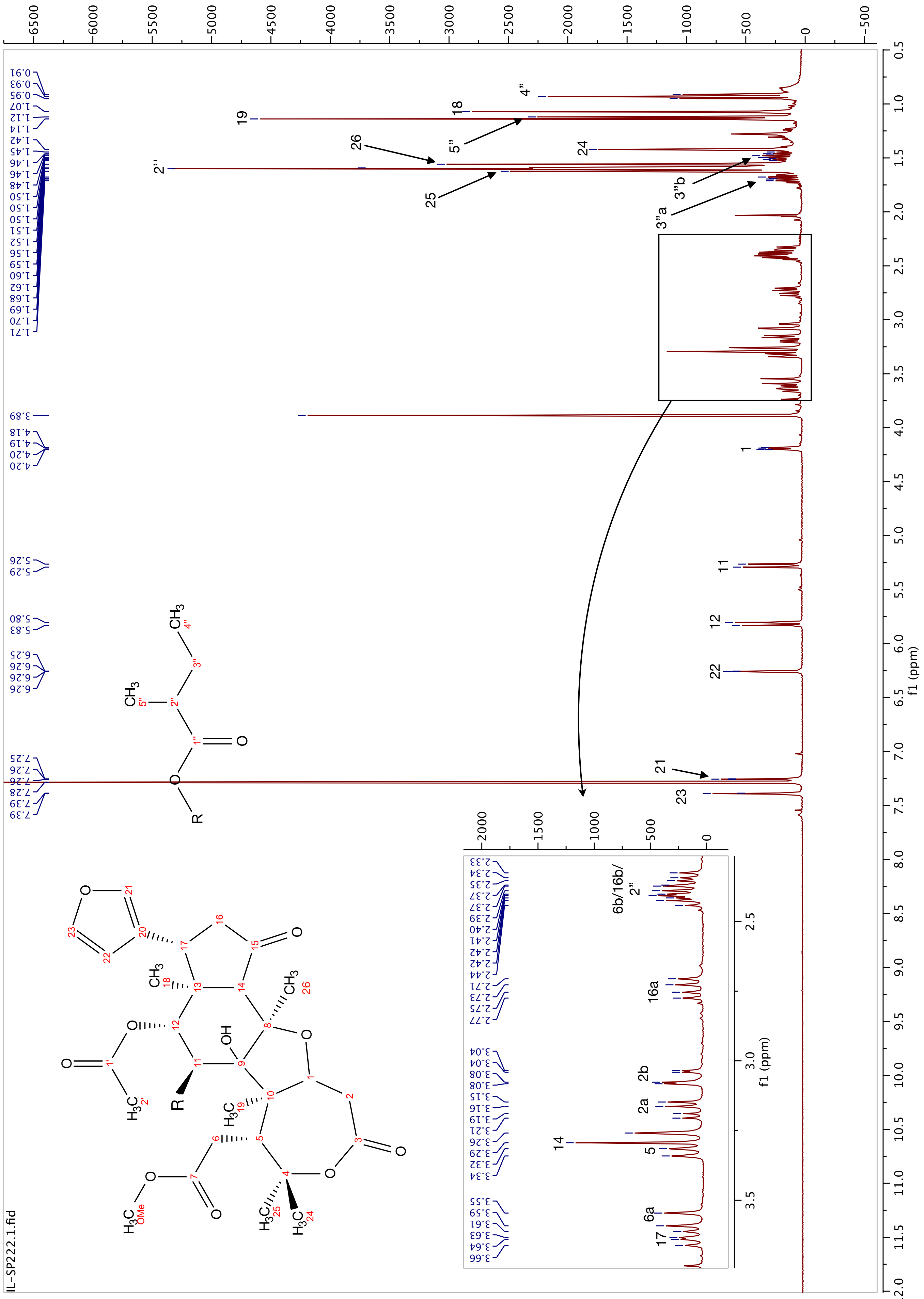

Figure S21: ^{13}C NMR of compound 3 ($CDCl_3$)

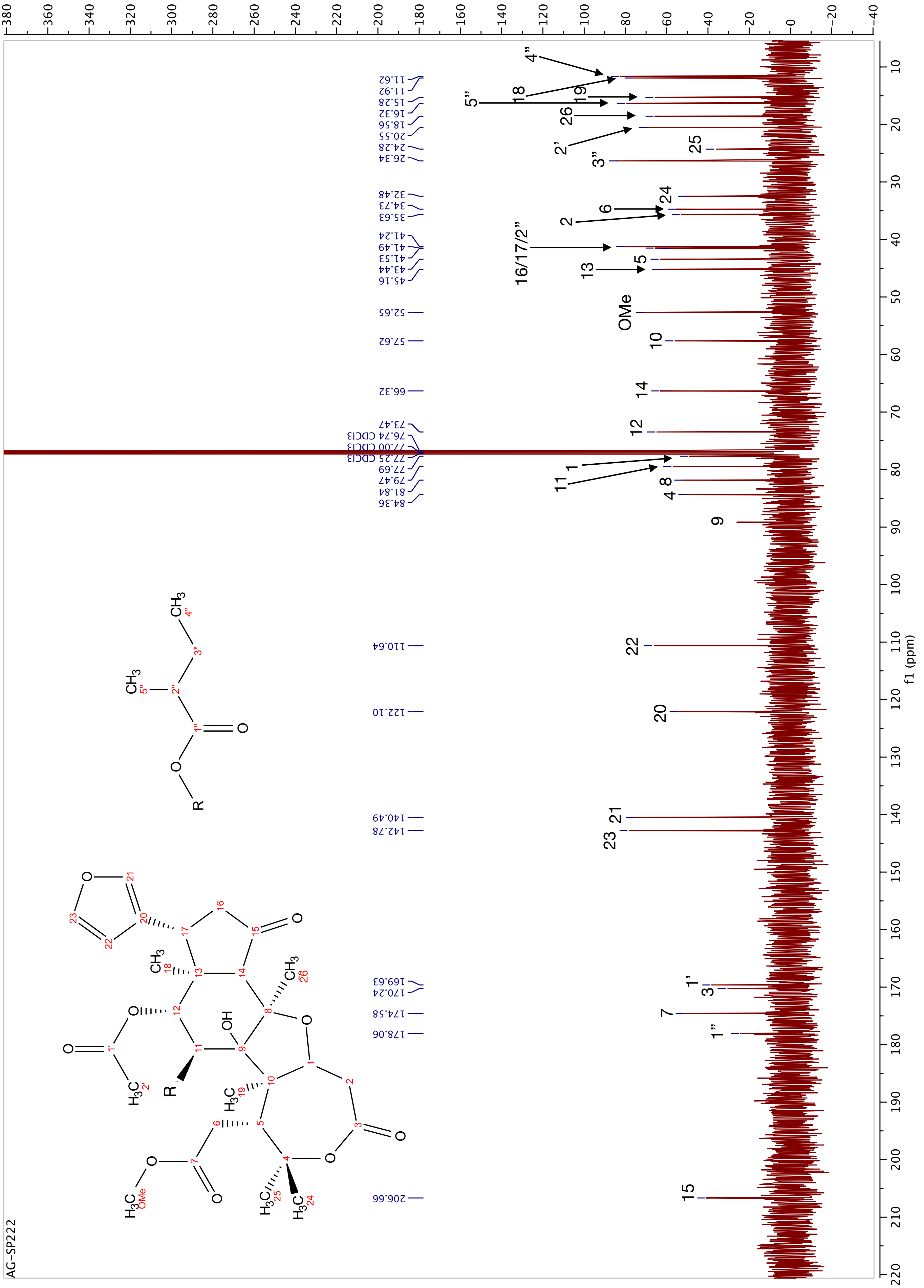

Figure S22: COSY experiment of compound 3 ($CDCl_3$)

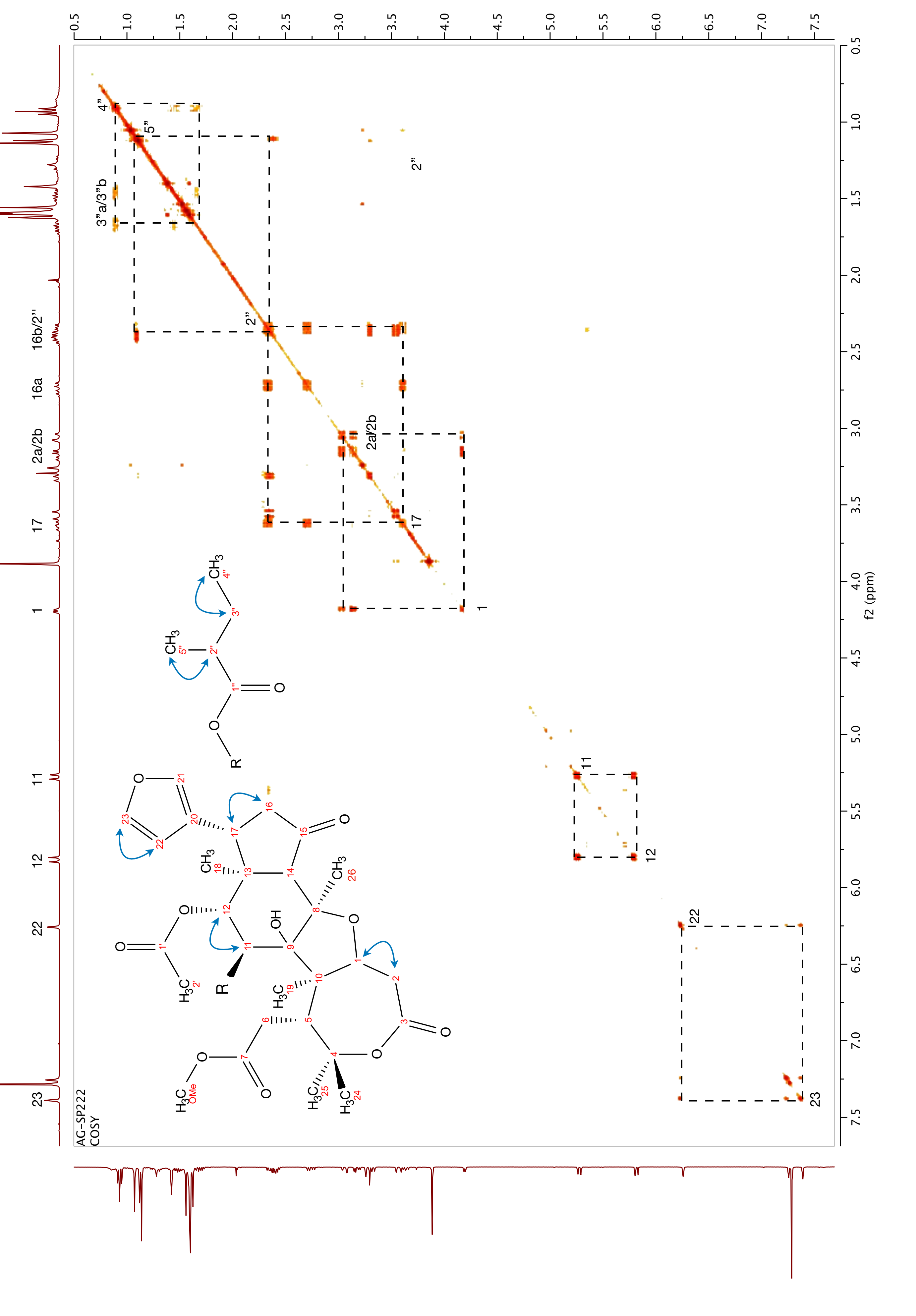

Figure S23: HMQC experiment of compound 3 (CDCl3)

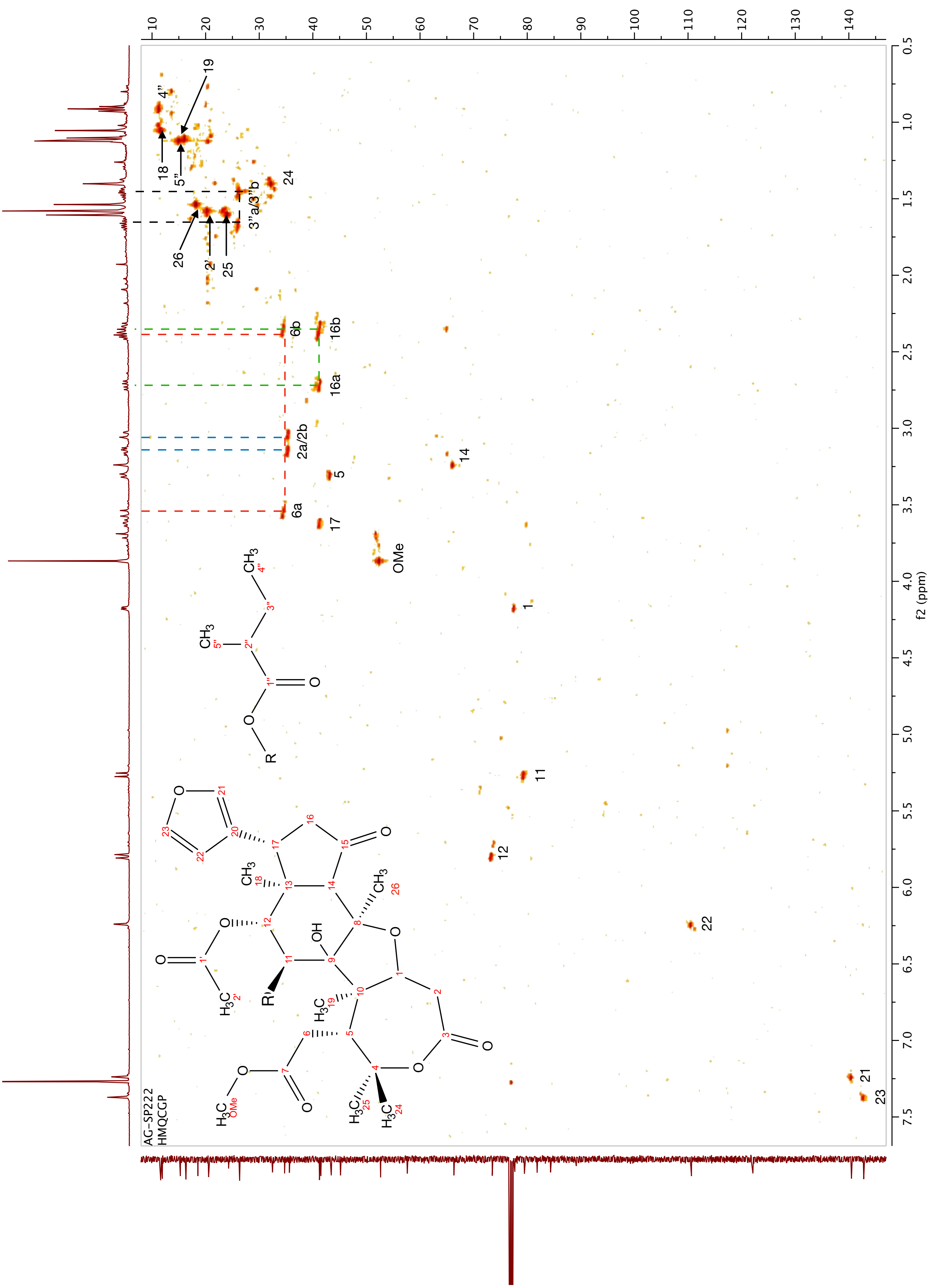

Figure S24: HMBC experiment of compound 3 ($CDCl_3$)

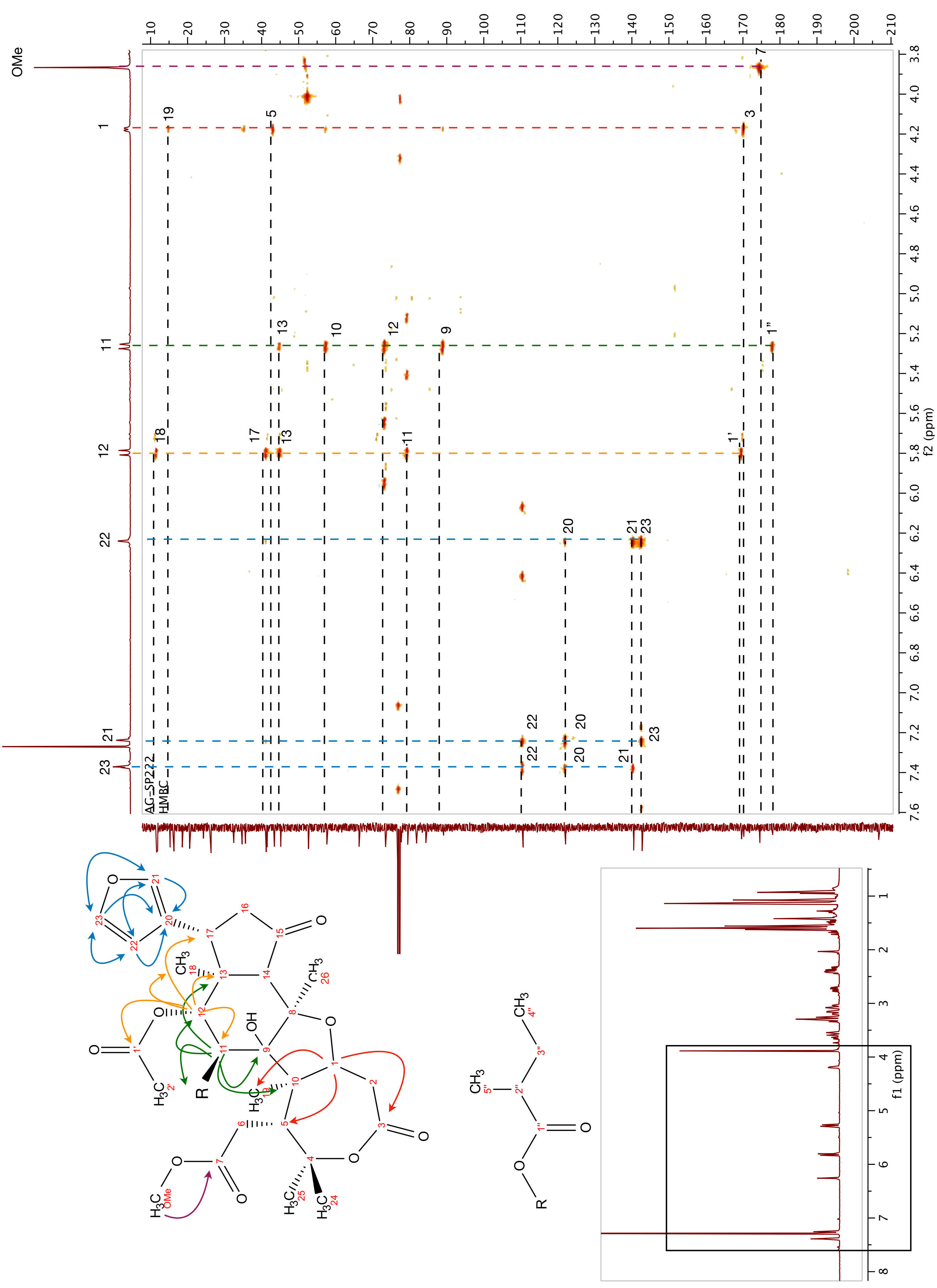

Figure S25: HMBC experiment of compound 3 ($CDCl_3$)

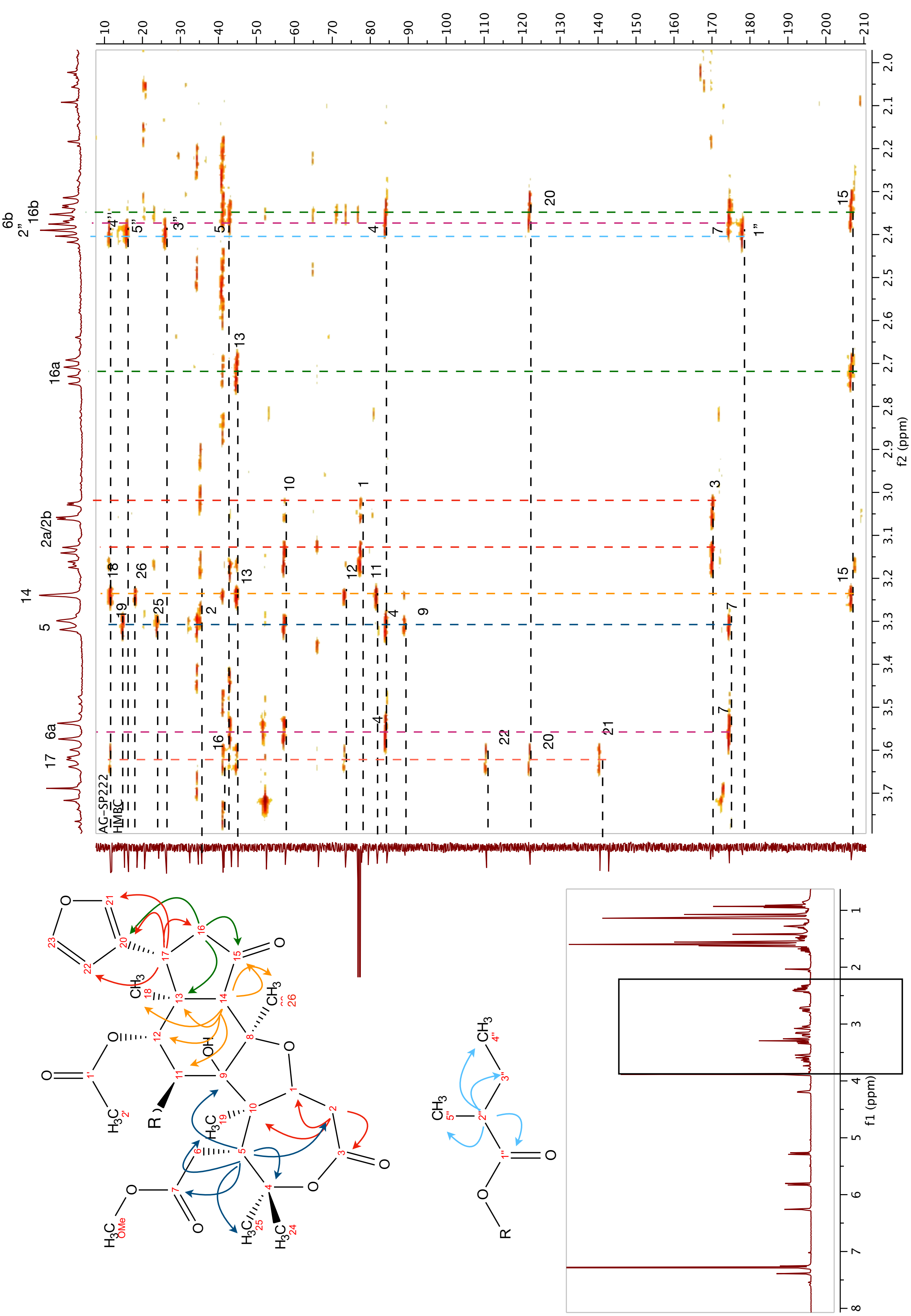

Figure S26: HMBC experiment of compound 3 ($CDCl_3$)

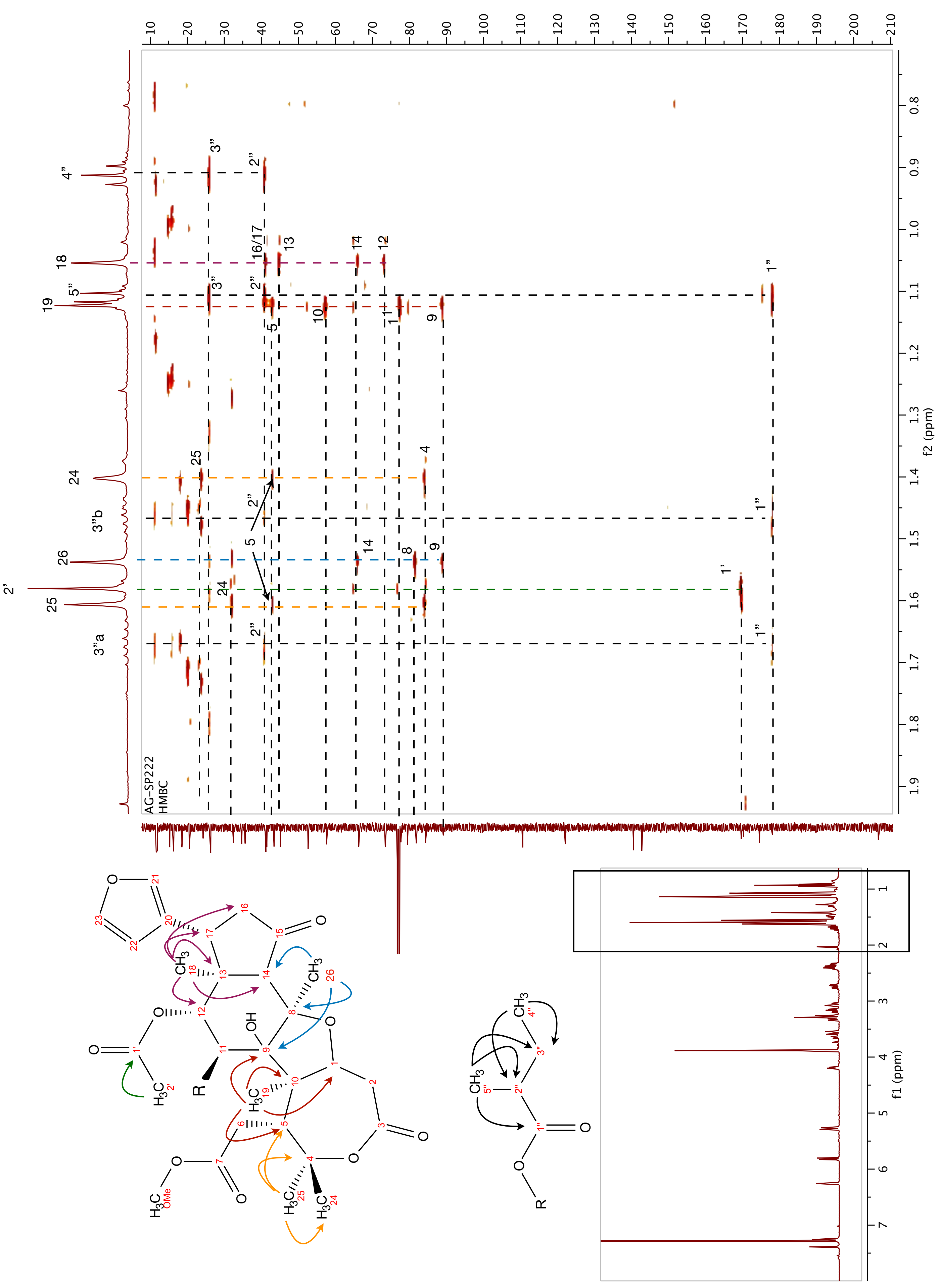

Figure S27: NOESY experiment of compound 3 ($CDCl_3$)

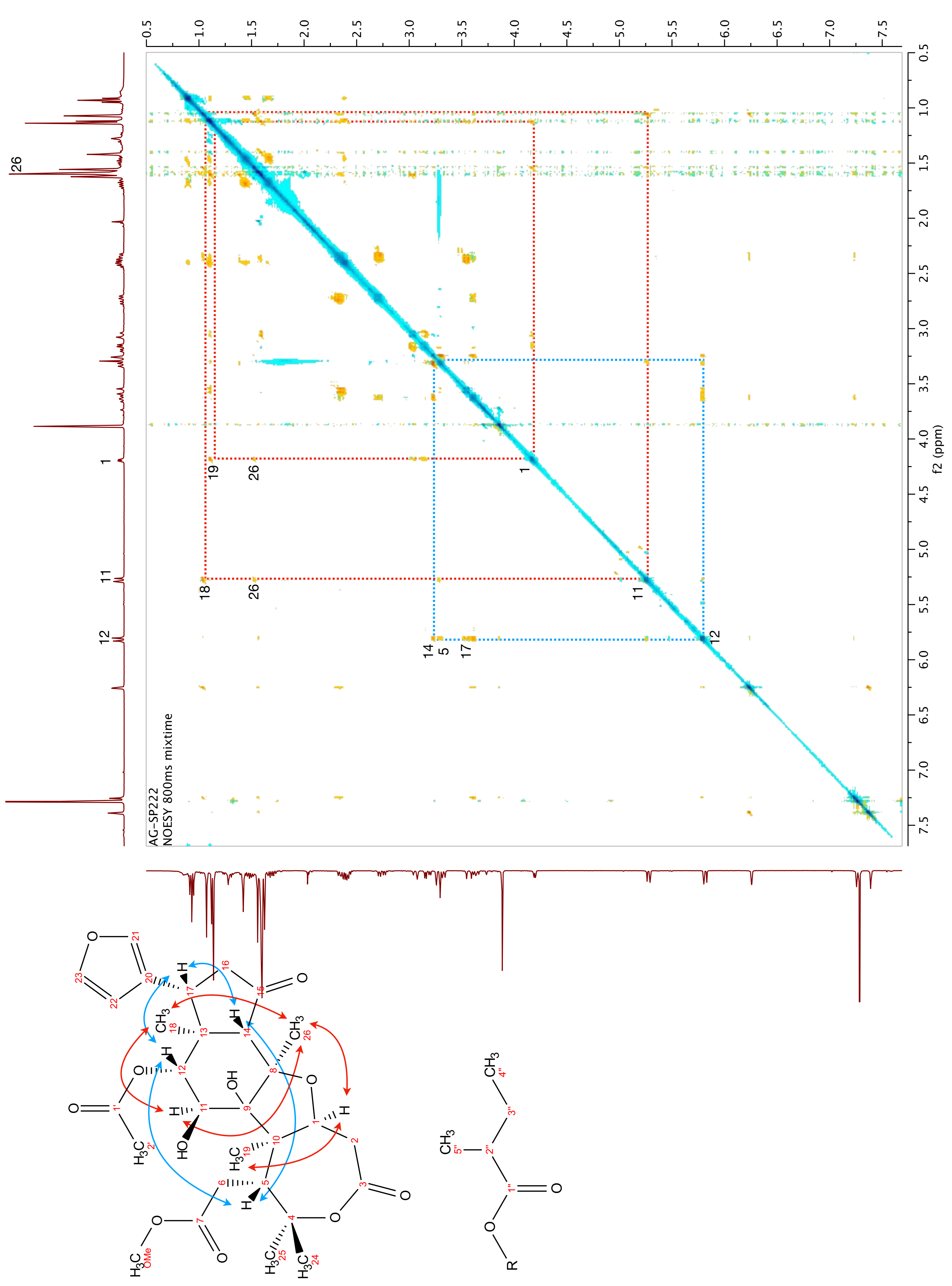

Figure S28: HRMS and IR of compound 3

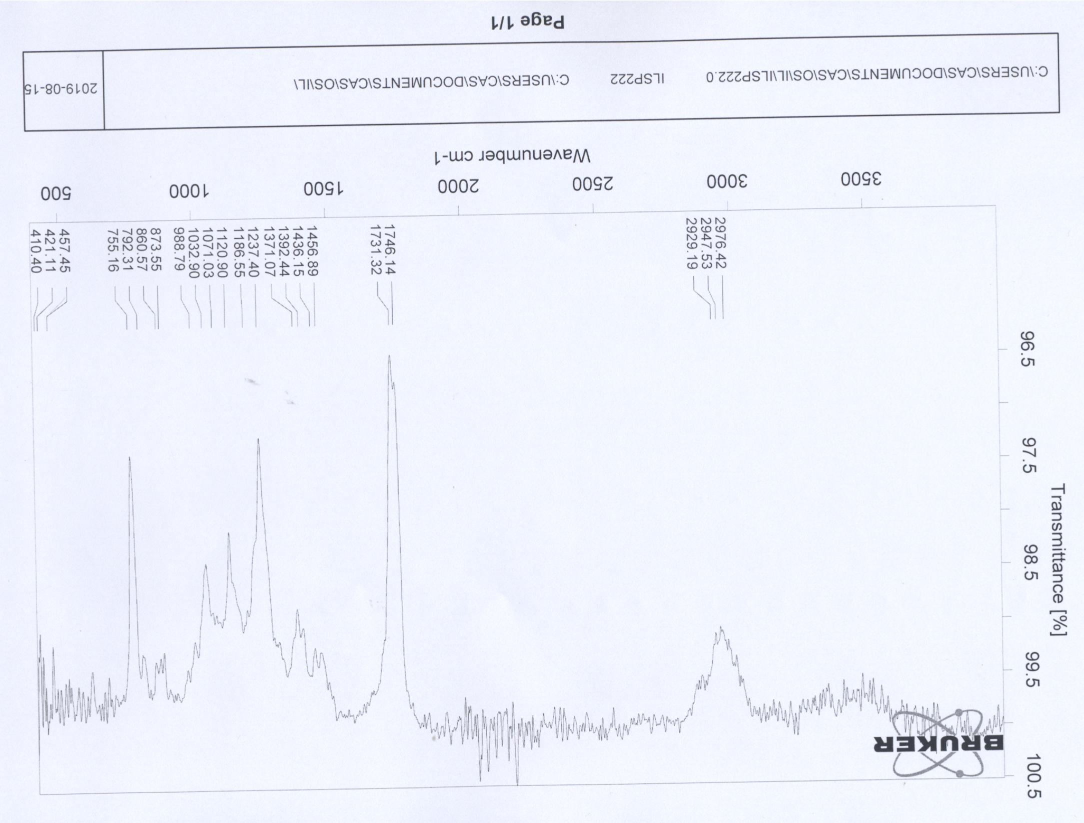

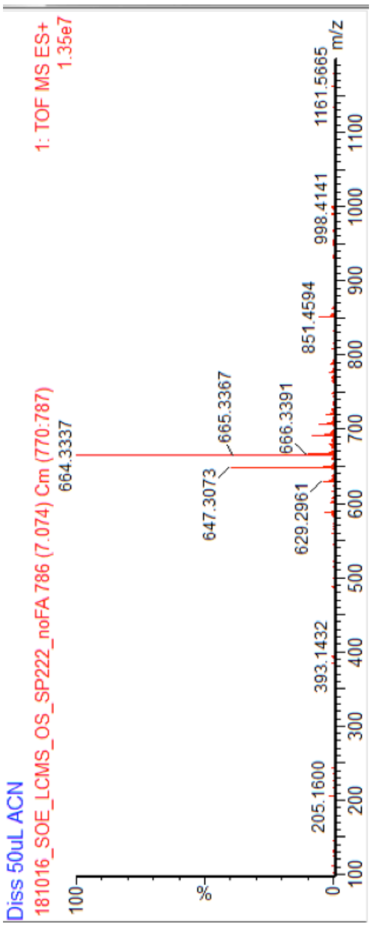

Single Mass Analysis
Tolerance = 2.0 mDa / DBE: min = -1.5, max = 100.0
Element prediction: Off
Number of isotope peaks used for i-FIT = 6

Monoisotopic Mass, Even Electron Ions
830 formula(e) evaluated with 4 results within limits (up to 50 closest results for each mass)
Elements Used:
C: 0-50 H: 0-100 N: 0-2 O: 0-20 Si: 0-1

Mass	Calc. Mass	mDa	PPM	DBE	Formula	i-FIT	i-FIT Norm	Fit Conf %	C	H	N	O	Si
647.3073	647.3068	0.5	0.8	11.5	C34 H47 O12	114.8	0.038	96.32	34	47		12	
	647.3062	1.1	1.7	29.5	C47 H39 N2 O	118.1	3.334	3.57	47	39	2	1	
	647.3086	-1.3	-2.0	-1.5	C22 H51 N2 O19	121.7	6.877	0.10	22	51	2	19	
	647.3059	1.4	2.2	2.5	C25 H51 N2 O15 Si	123.8	9.010	0.01	25	51	2	15	1

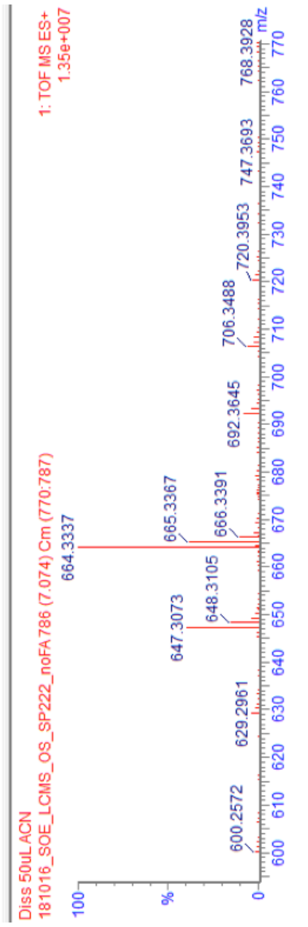

Figure S29: ¹H NMR of compound 4 ($CDCl_3$)

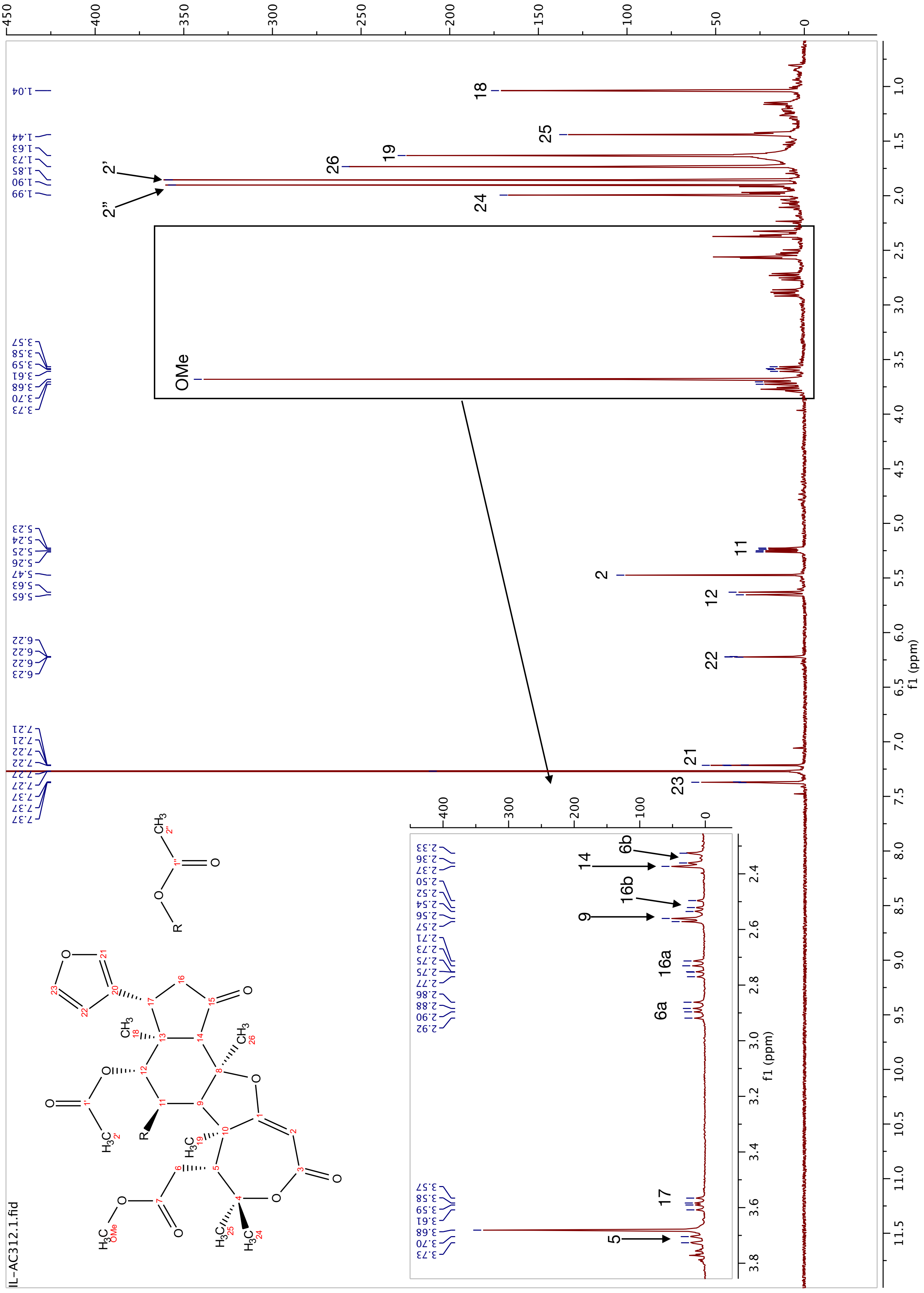

Figure S30: ^{13}C NMR of compound 4 ($CDCl_3$)

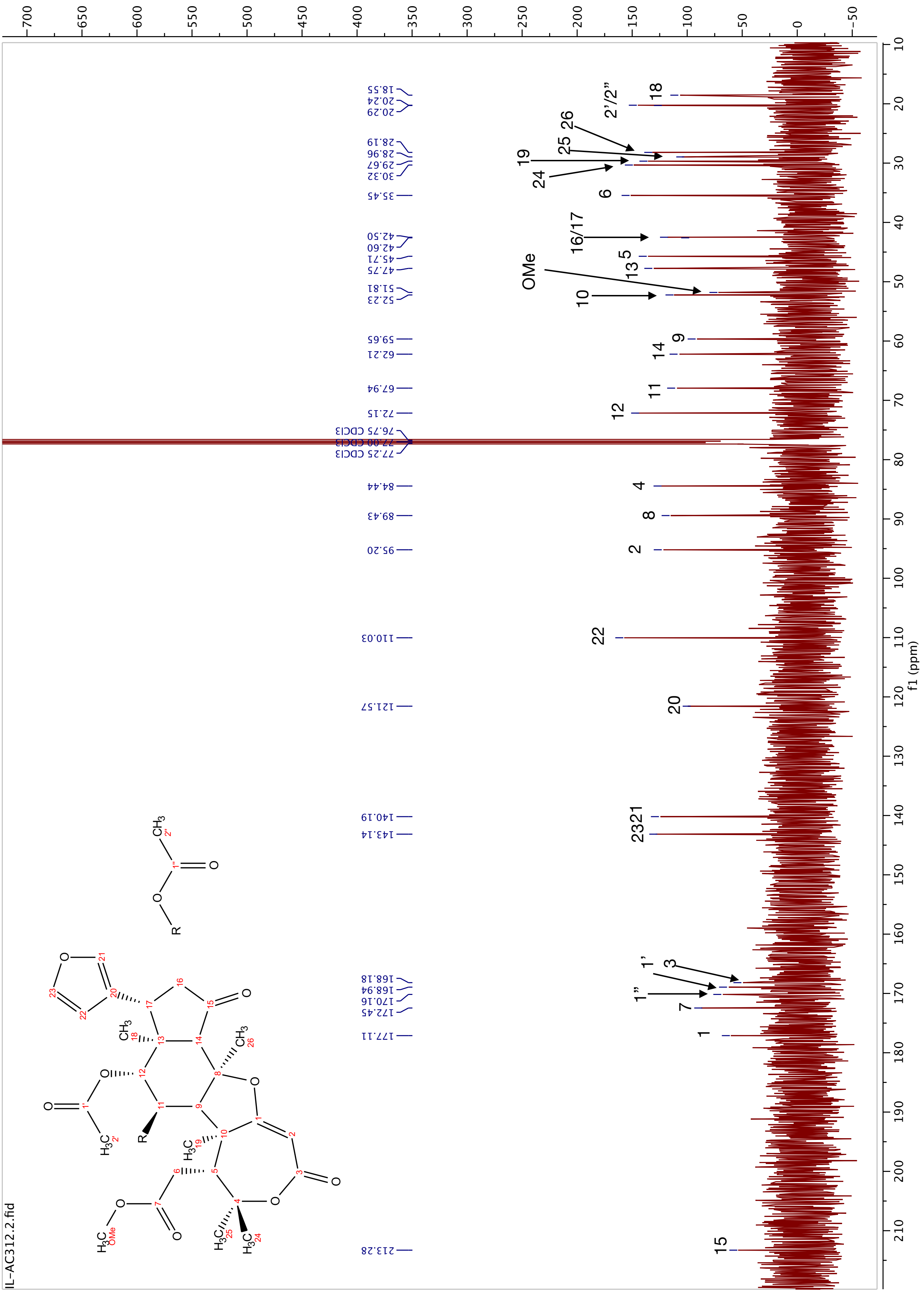

Figure S31: COSY experiment of compound 4 ($CDCl_3$)

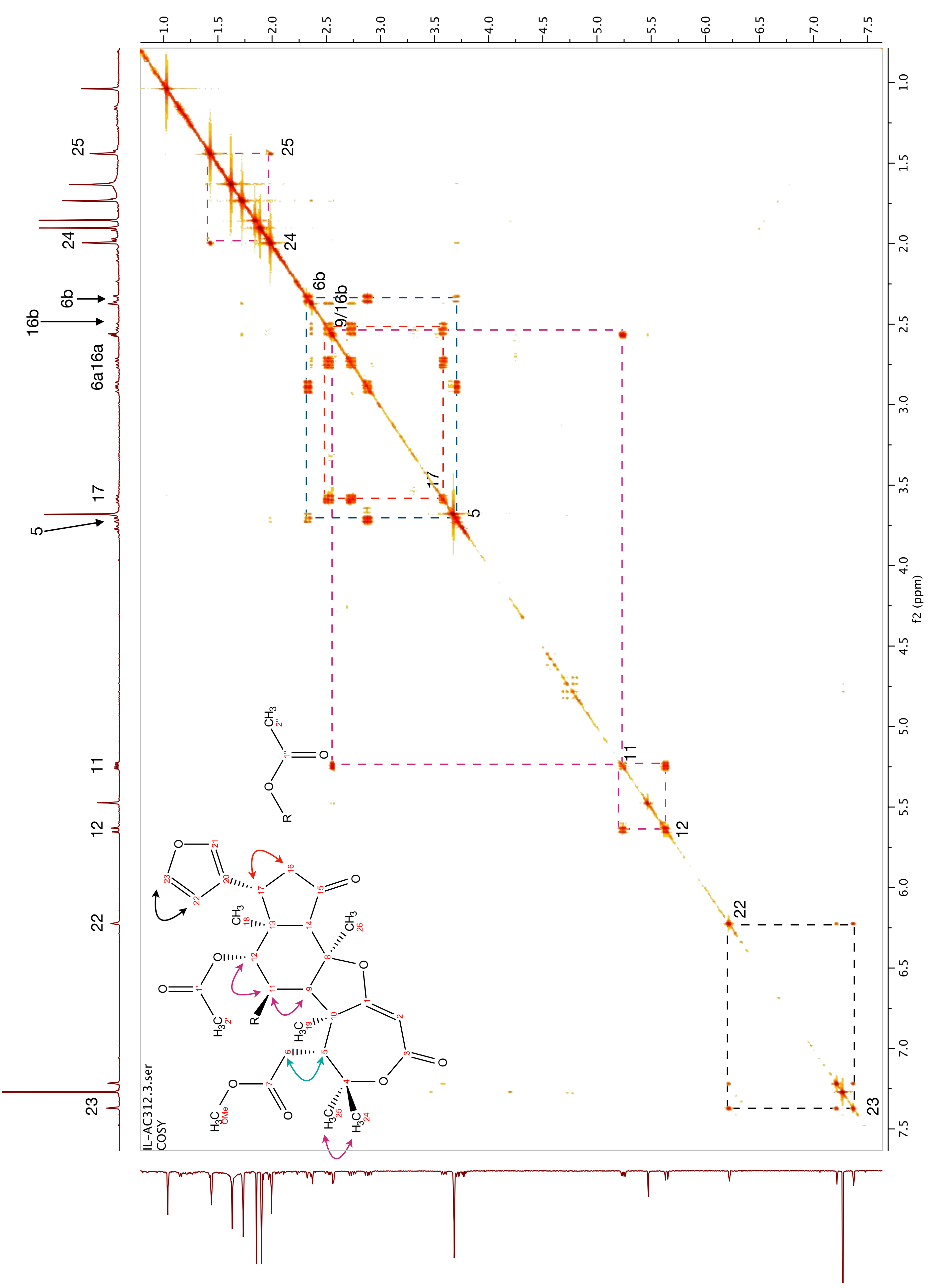

Figure S32: HMQC experiment of compound 4 (CDCl3)

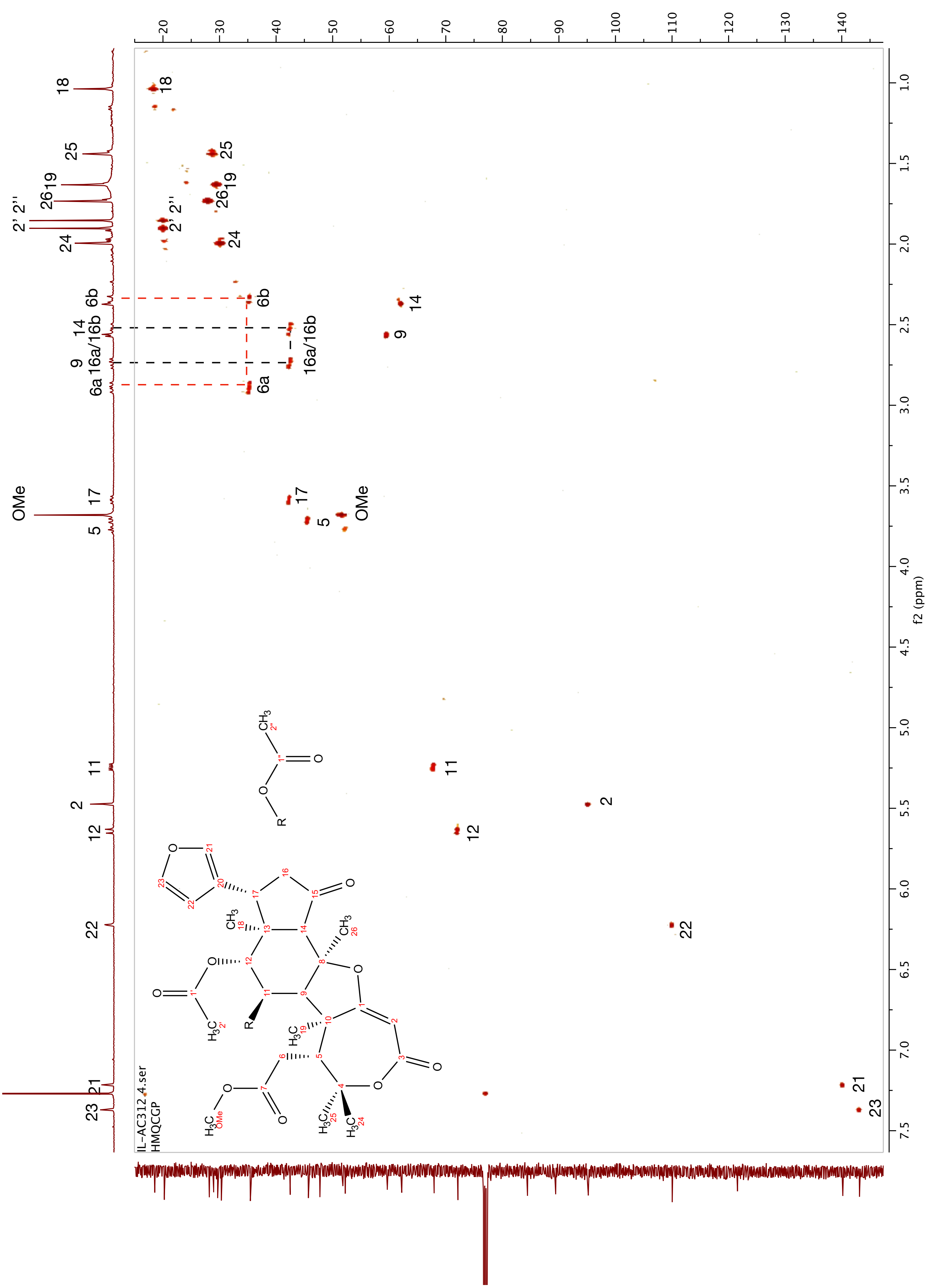

Figure S33: HMBC experiment of compound 4 ($CDCl_3$)

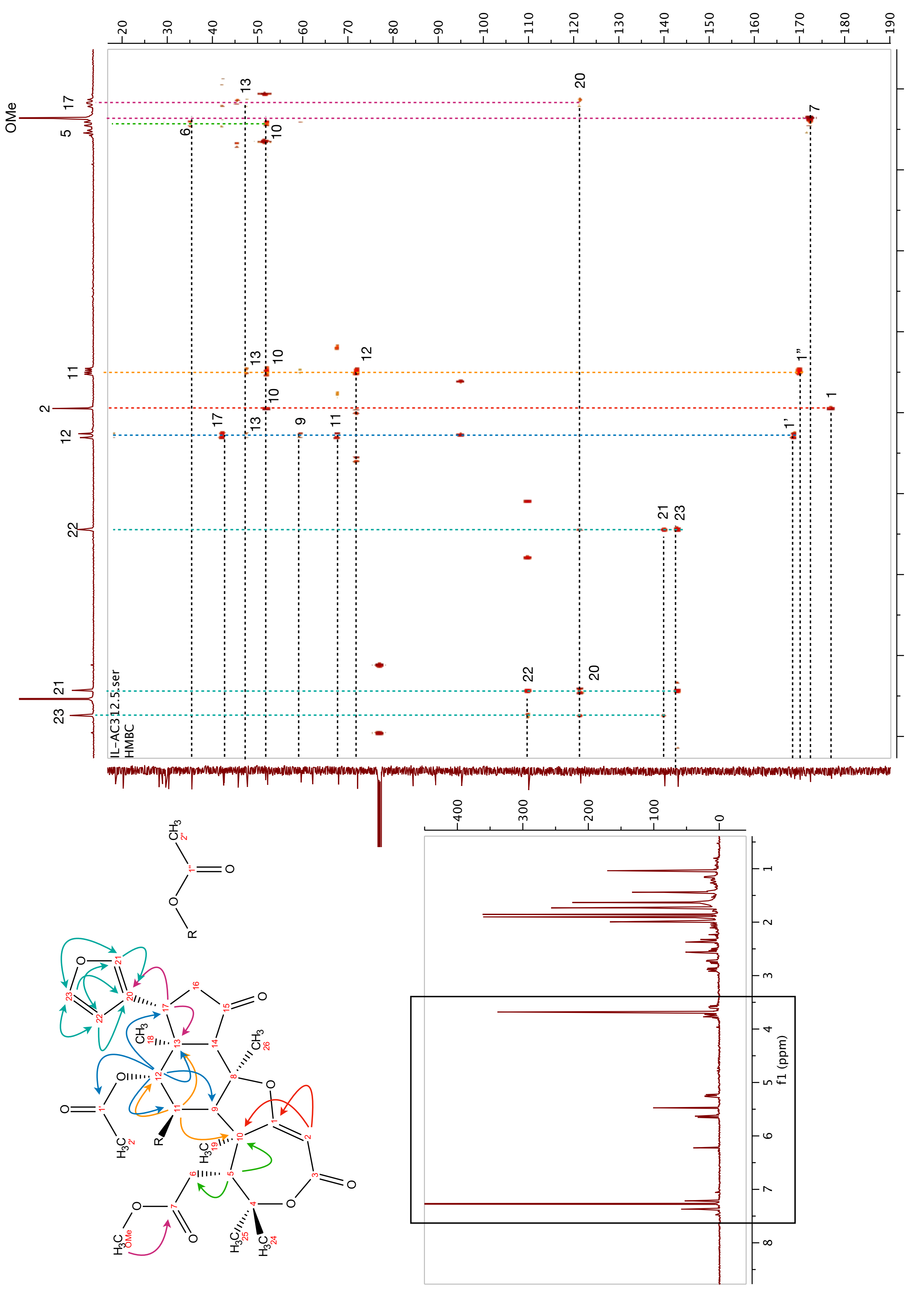

Figure S34: HMBC experiment of compound 4 ($CDCl_3$)

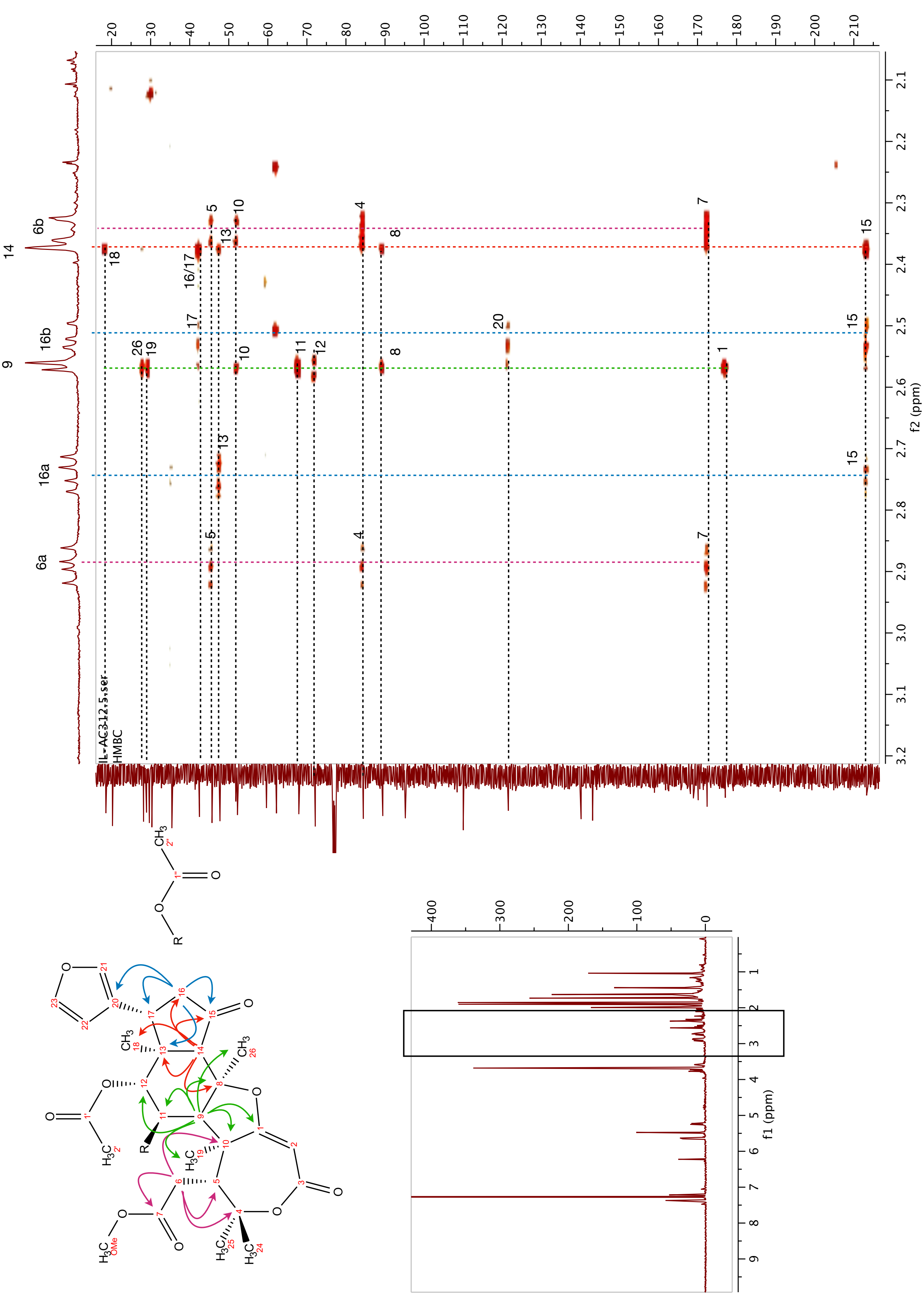

Figure S35: HMBC experiment of compound 4 ($CDCl_3$)

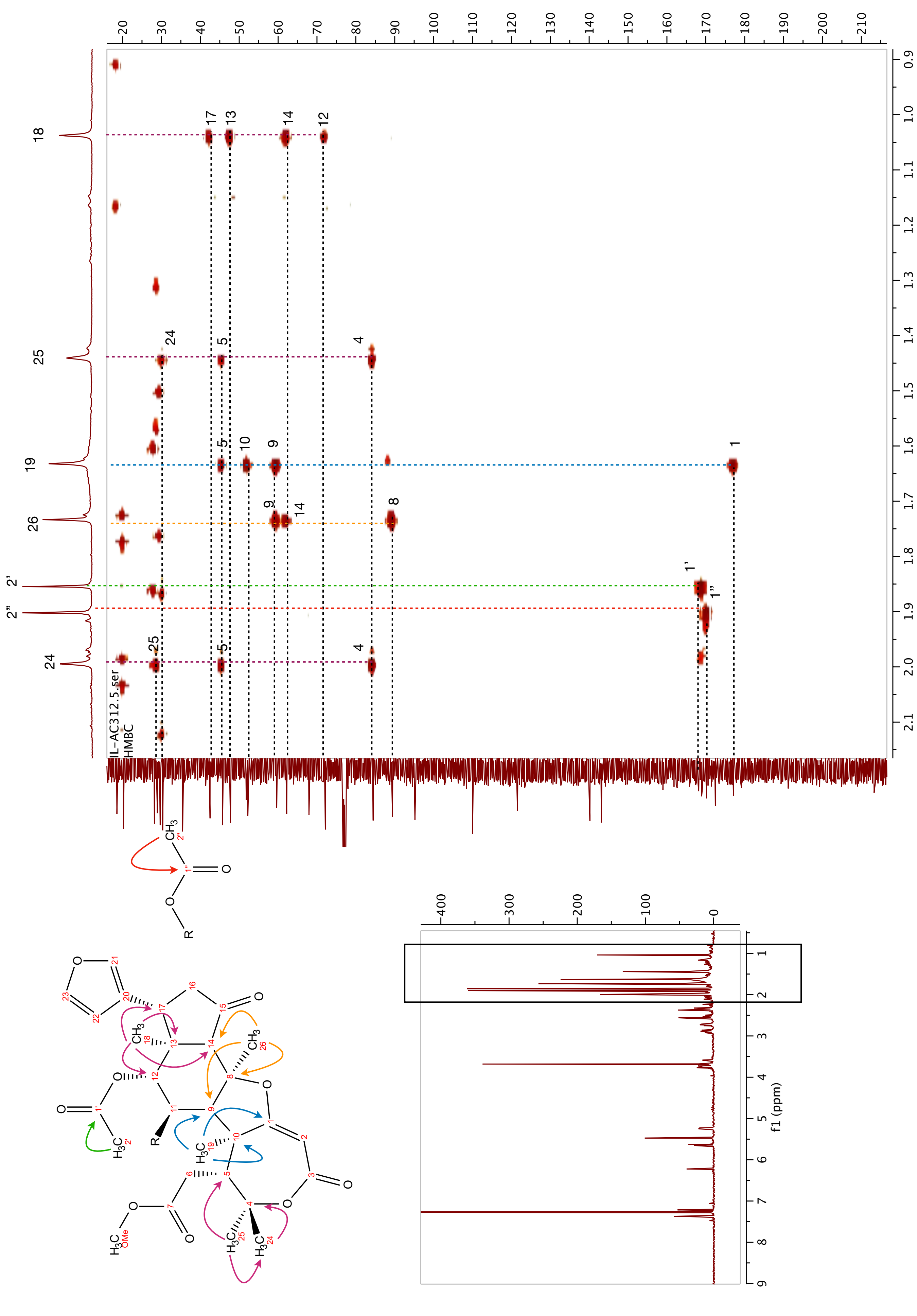

Figure S36: HRMS and IR spectra of compound 4

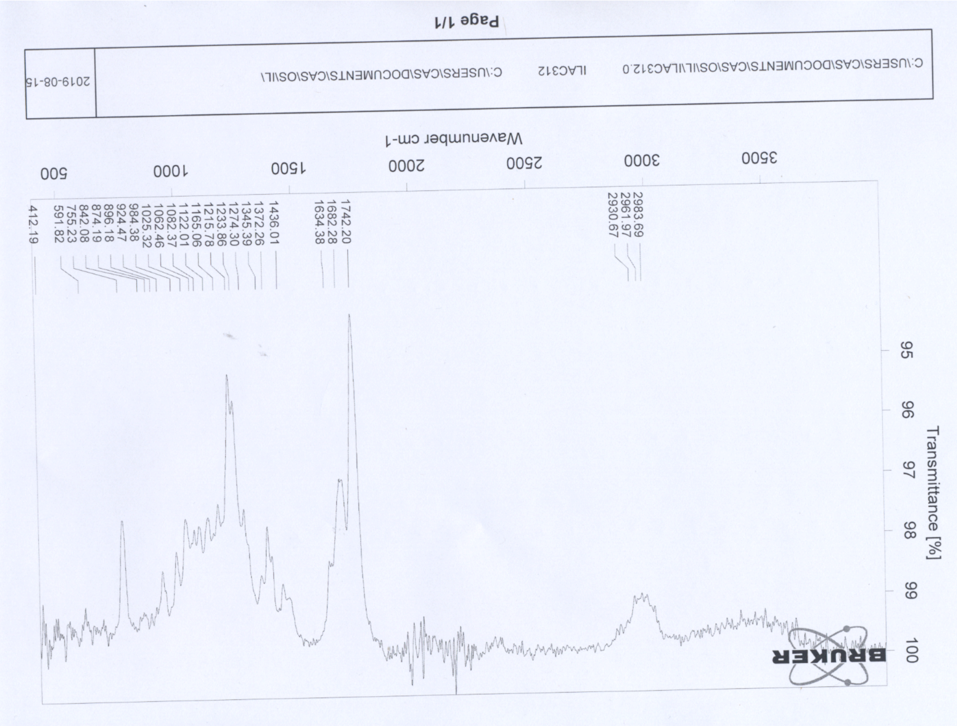

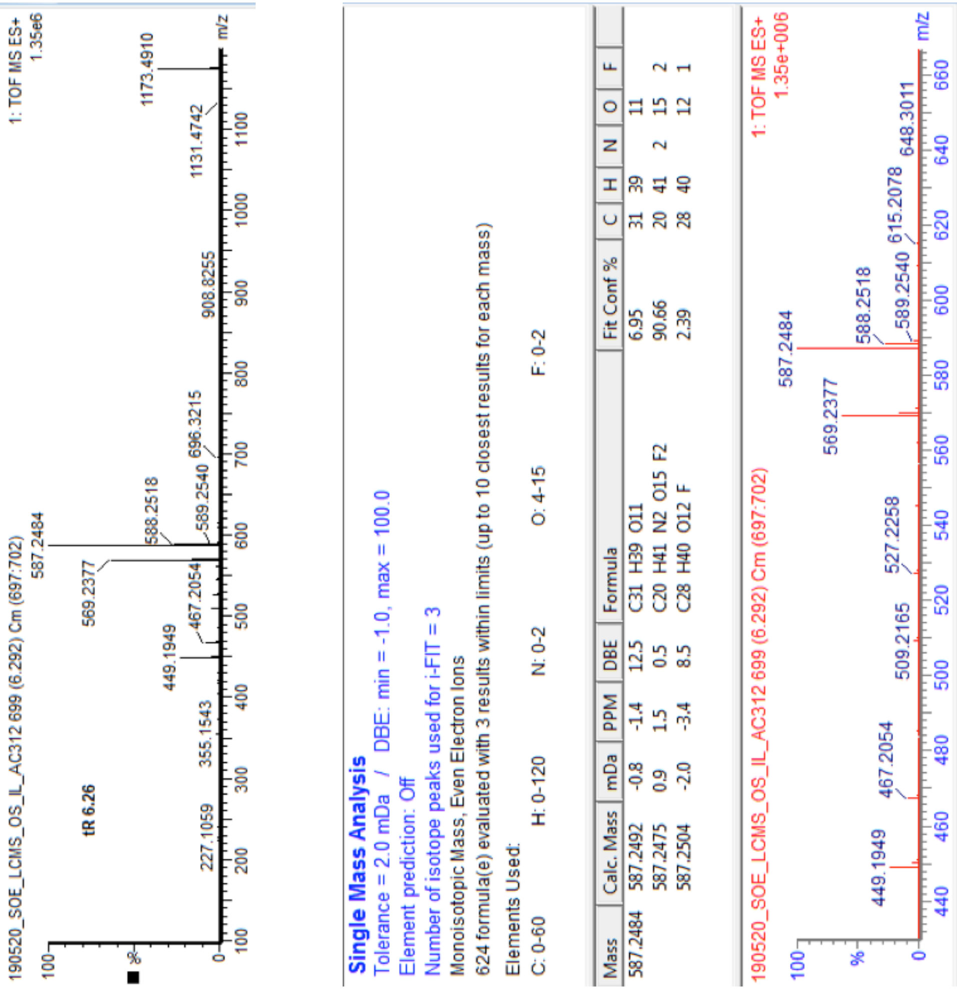

Figure S37: ^{1}H NMR of compound 5 ($CDCl_3$)

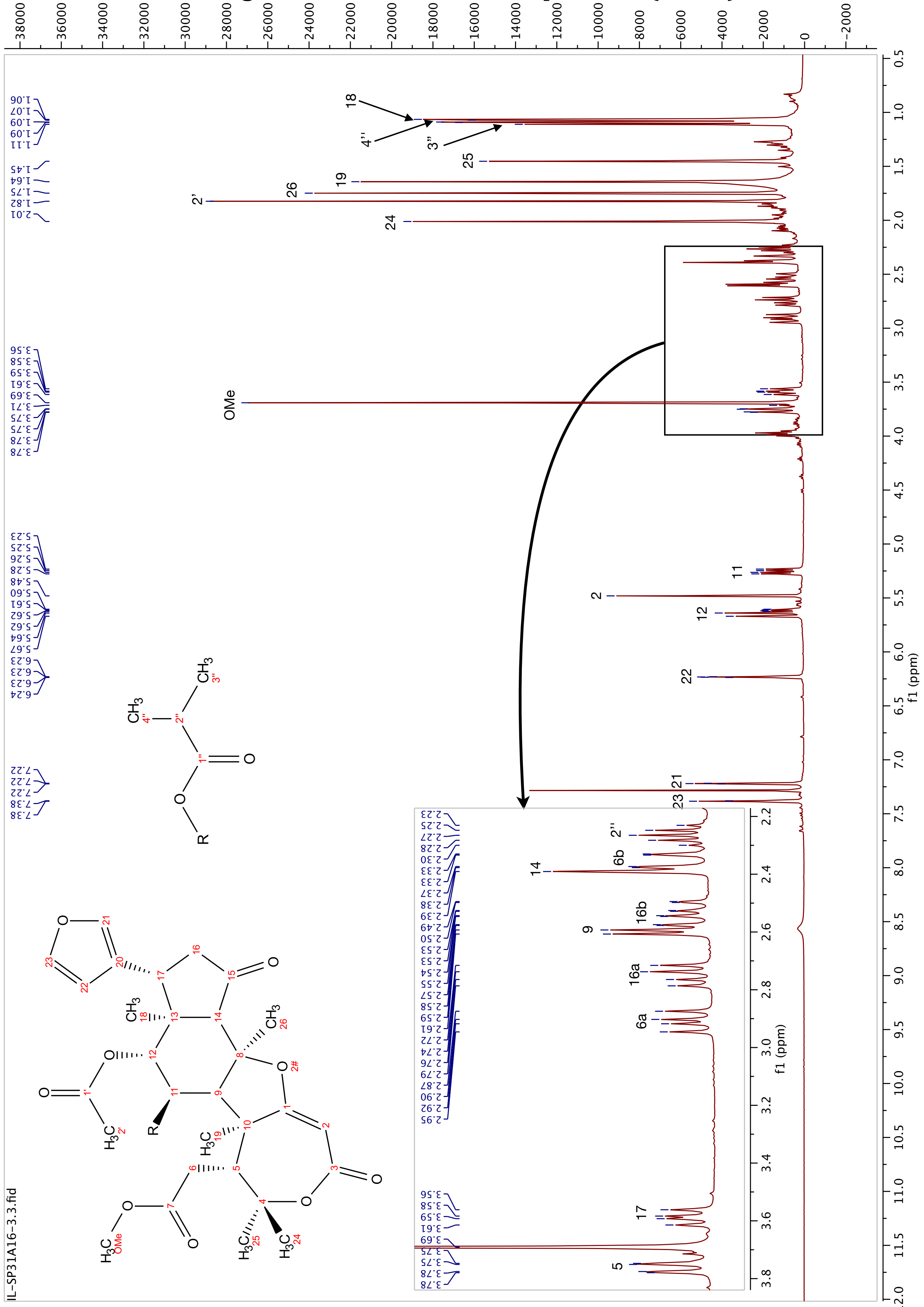

Figure S38: ^{13}C NMR of compound 5 ($CDCl_3$)

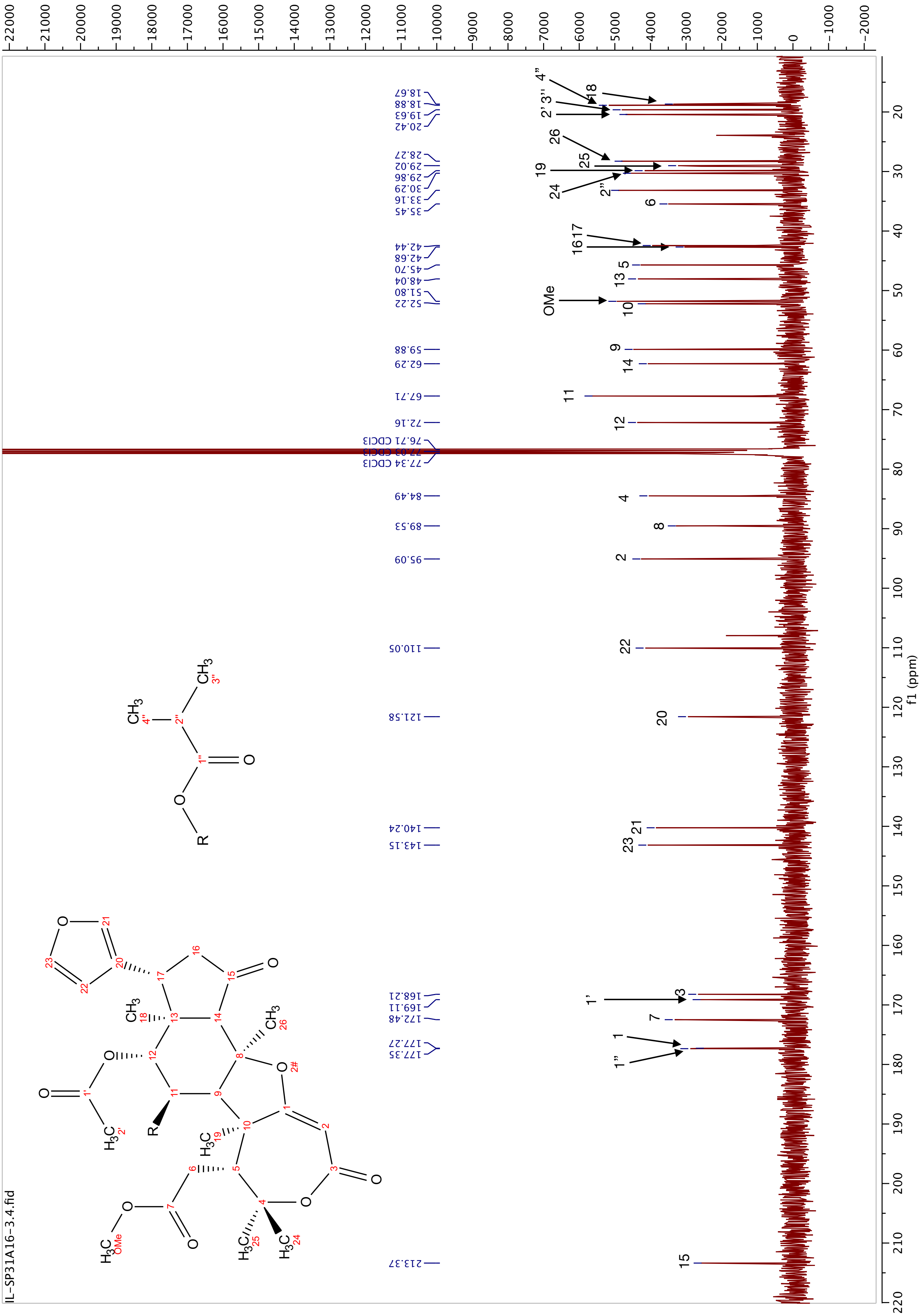

Figure S39: ^{13}C and DEPT135 spectra of compound 5 ($CDCl_3$)

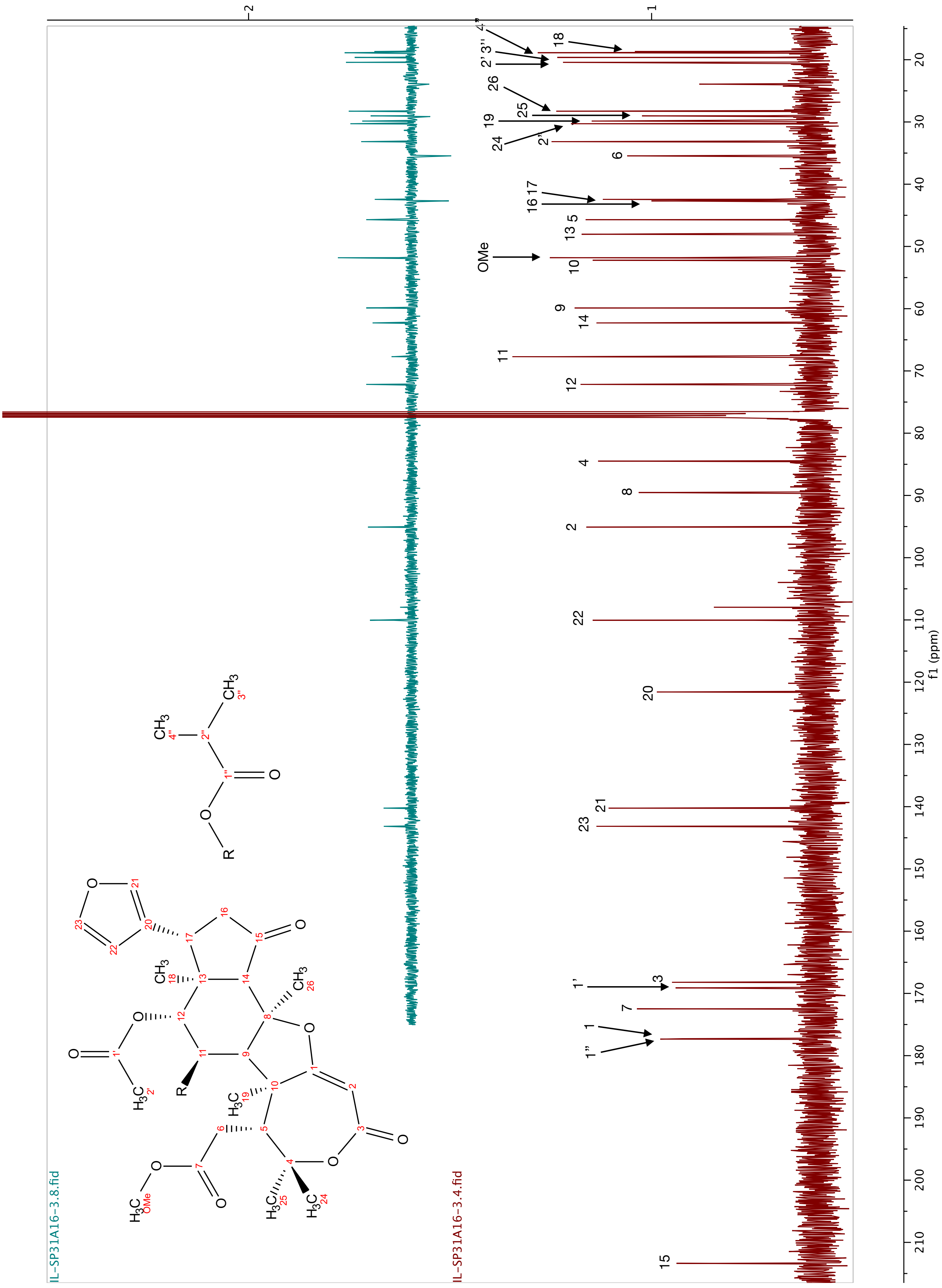

Figure S40: COSY experiment of compound 5 ($CDCl_3$)

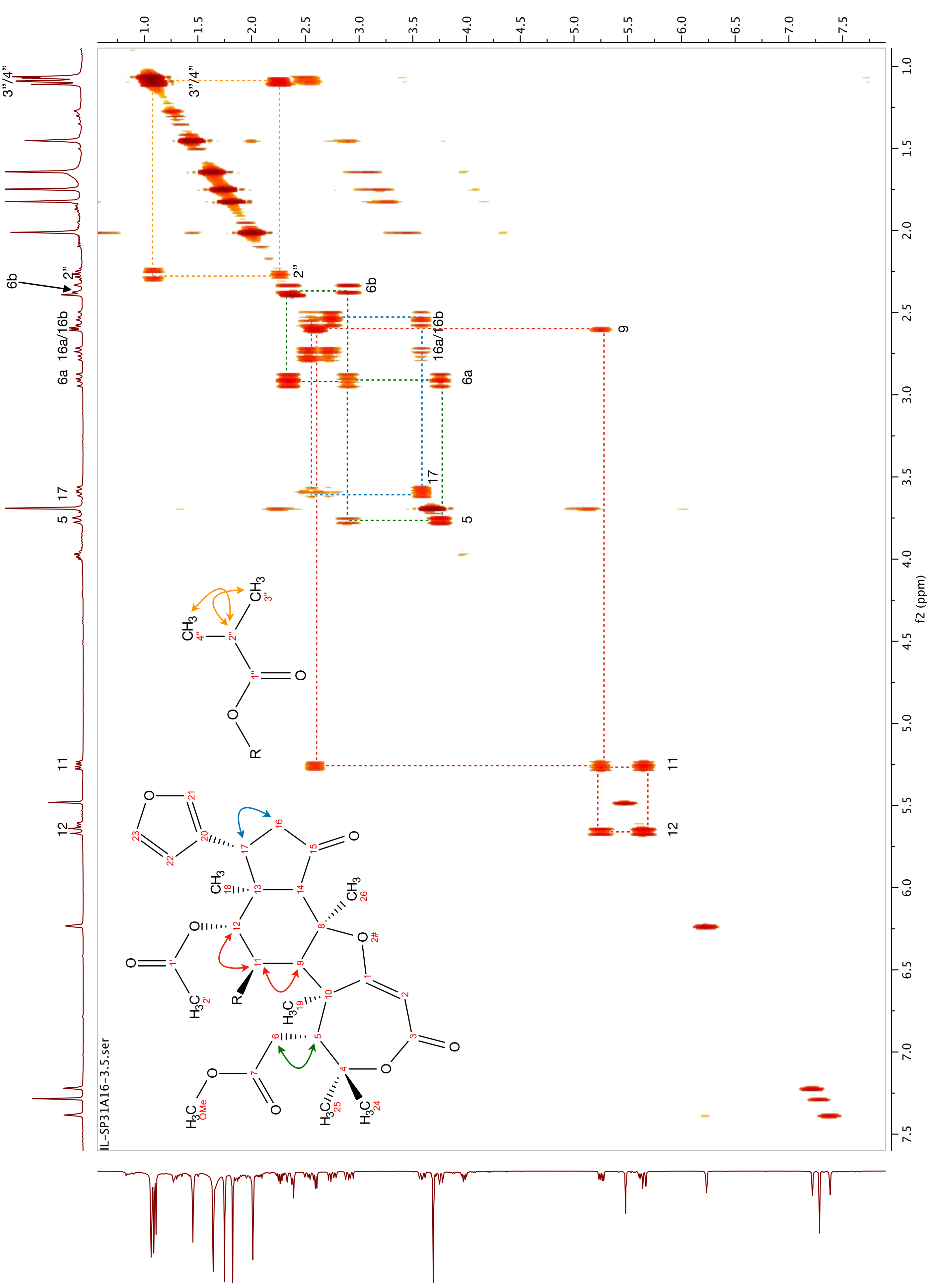

Figure S41: HMQC experiment of compound 5 (CDCl3)

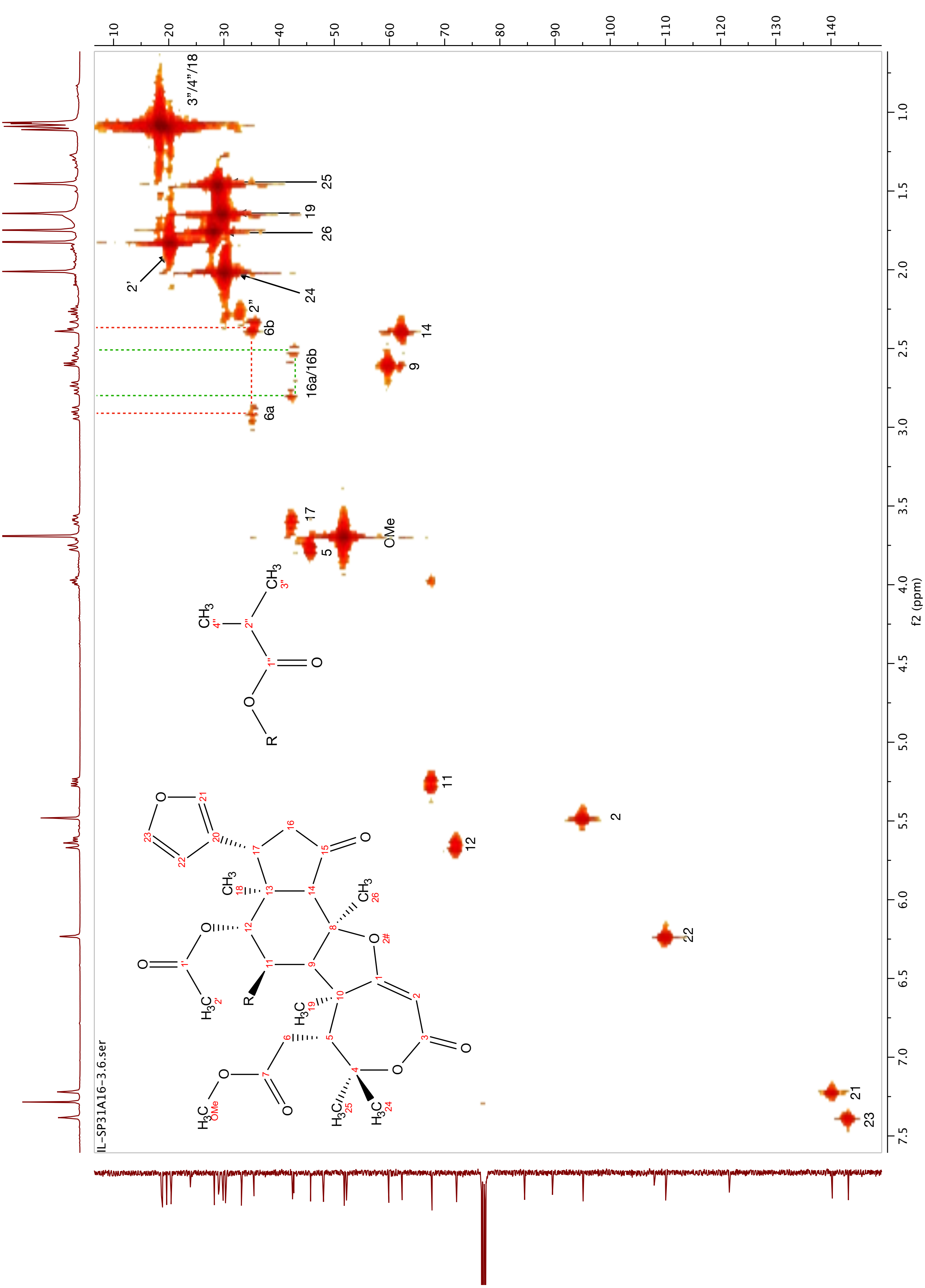

Figure S42: HMBC experiment of compound 5 ($CDCl_3$)

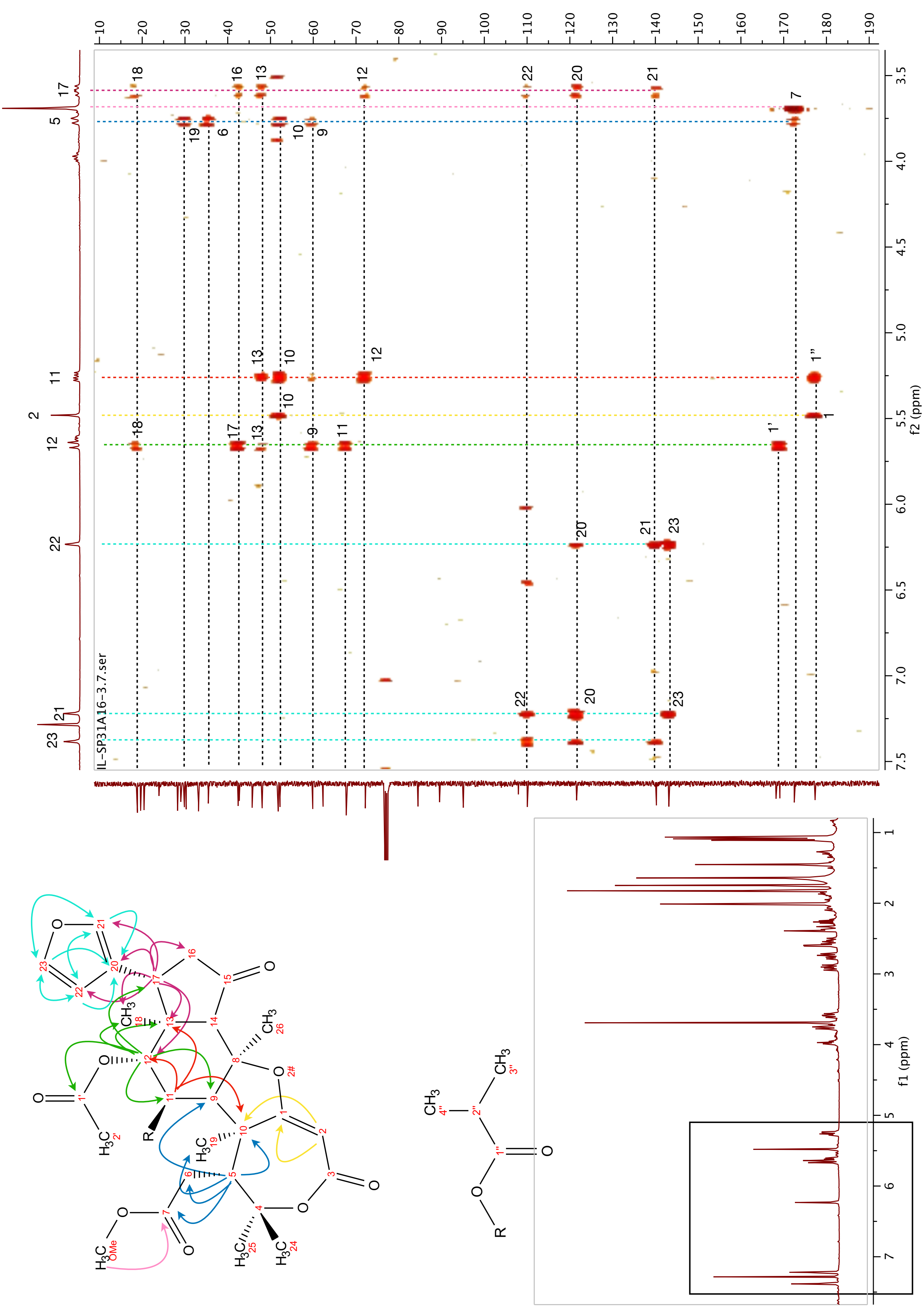

Figure S43: HMBC experiment of compound 5 ($CDCl_3$)

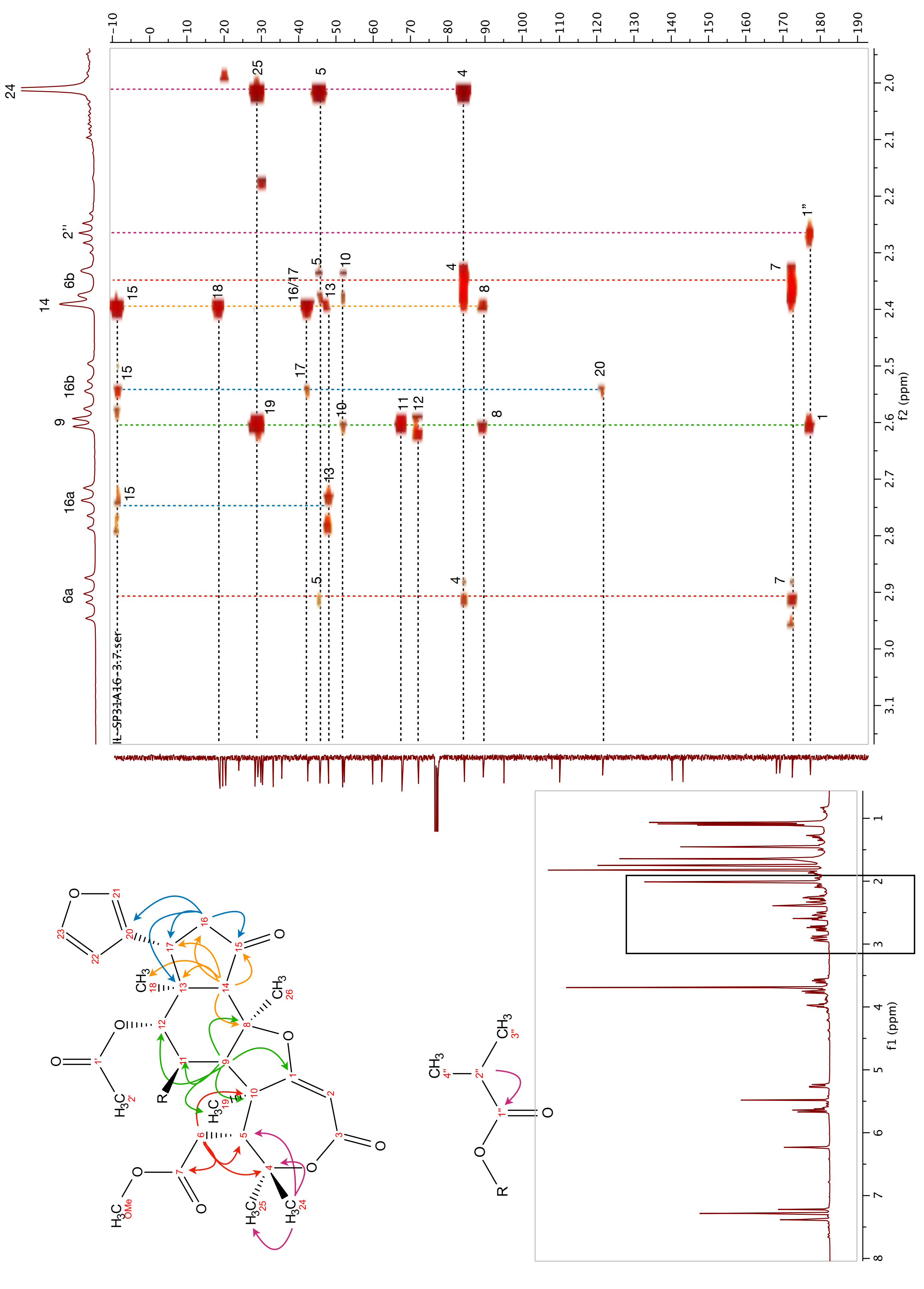

Figure S44: HMBC experiment of compound 5 (CDCl$_3$)

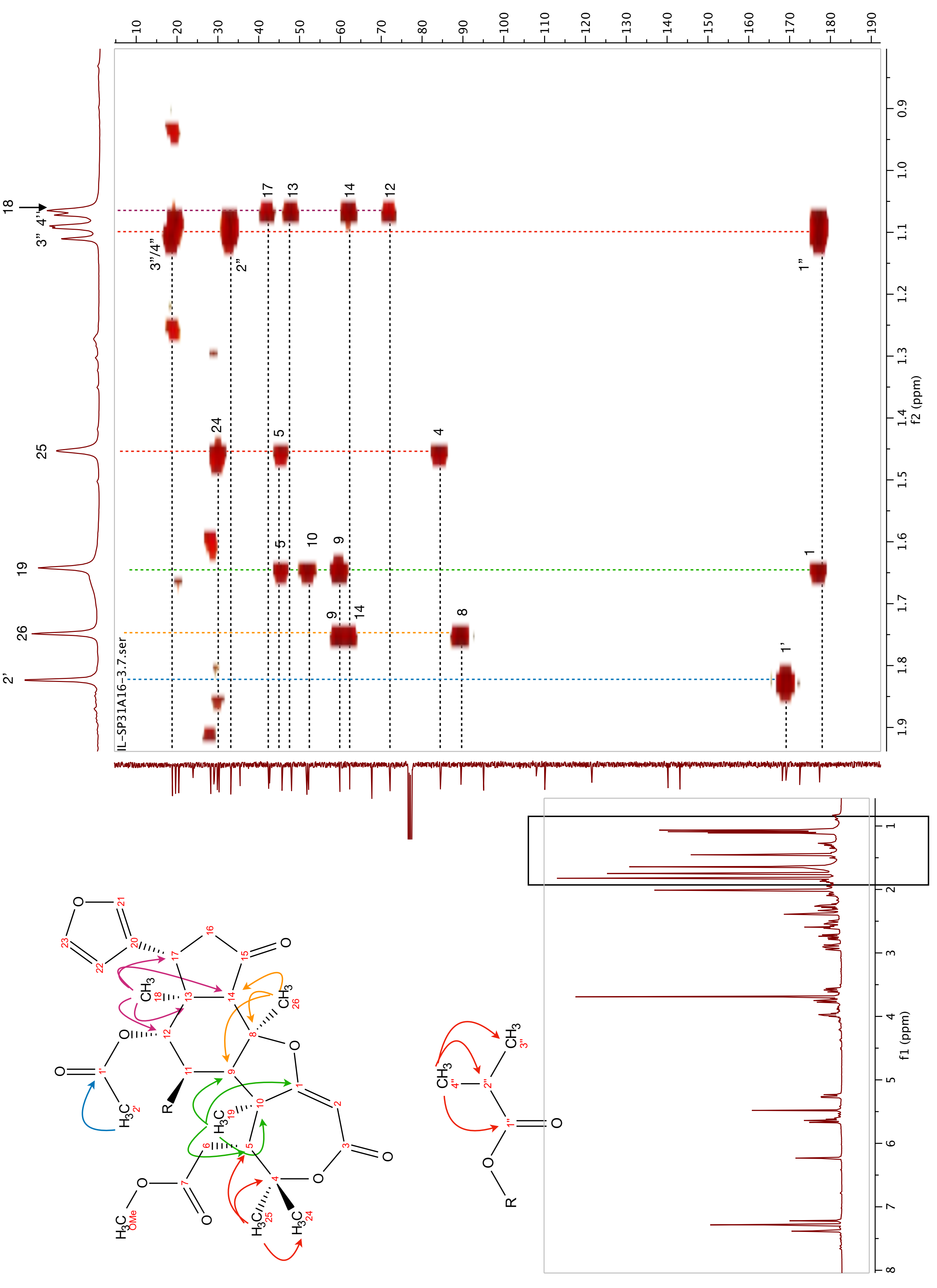

Figure S45: NOESY experiment of compound 5 ($CDCl_3$)

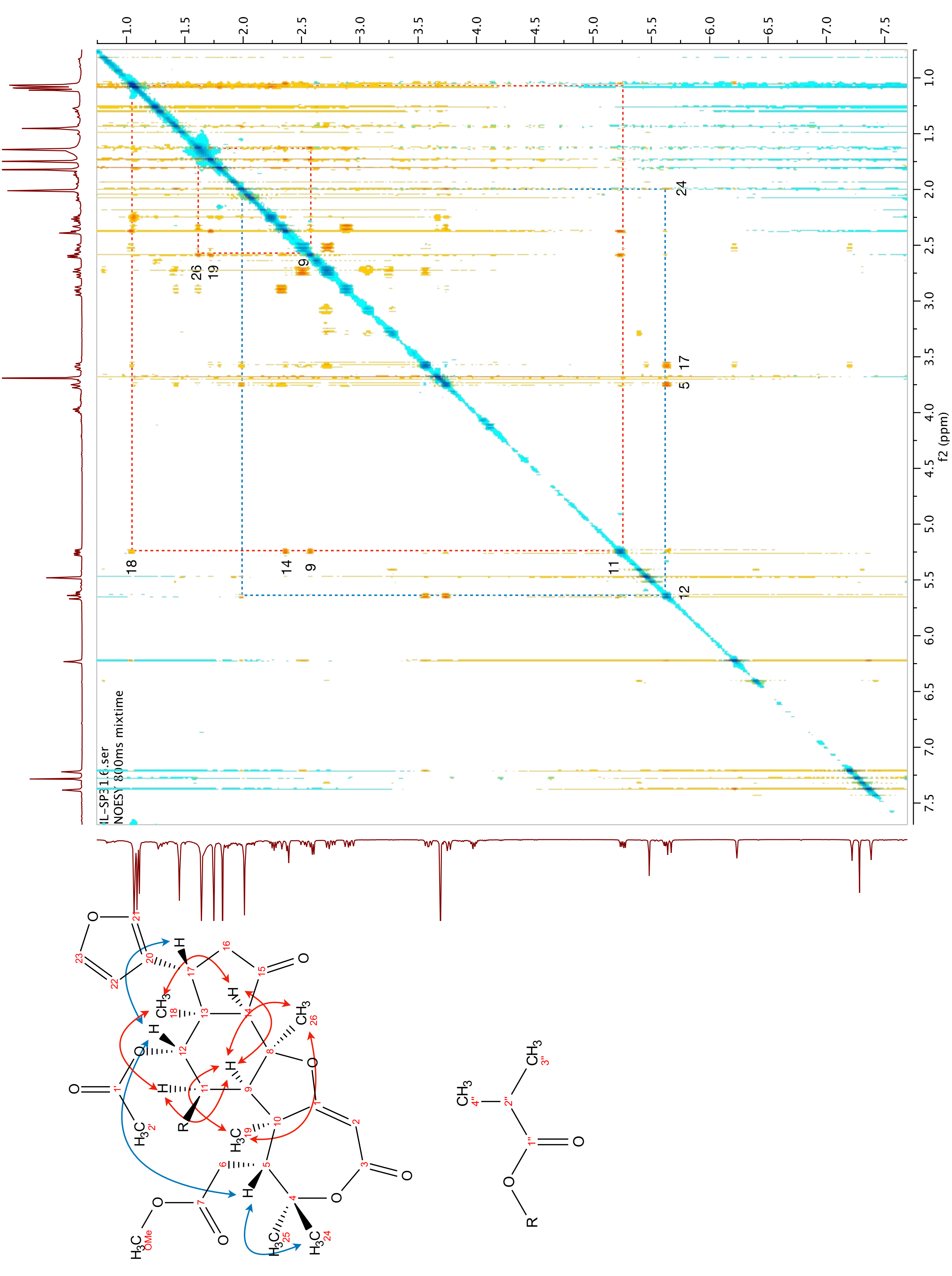

Figure S46: HRMS and IR spectra of compound 5

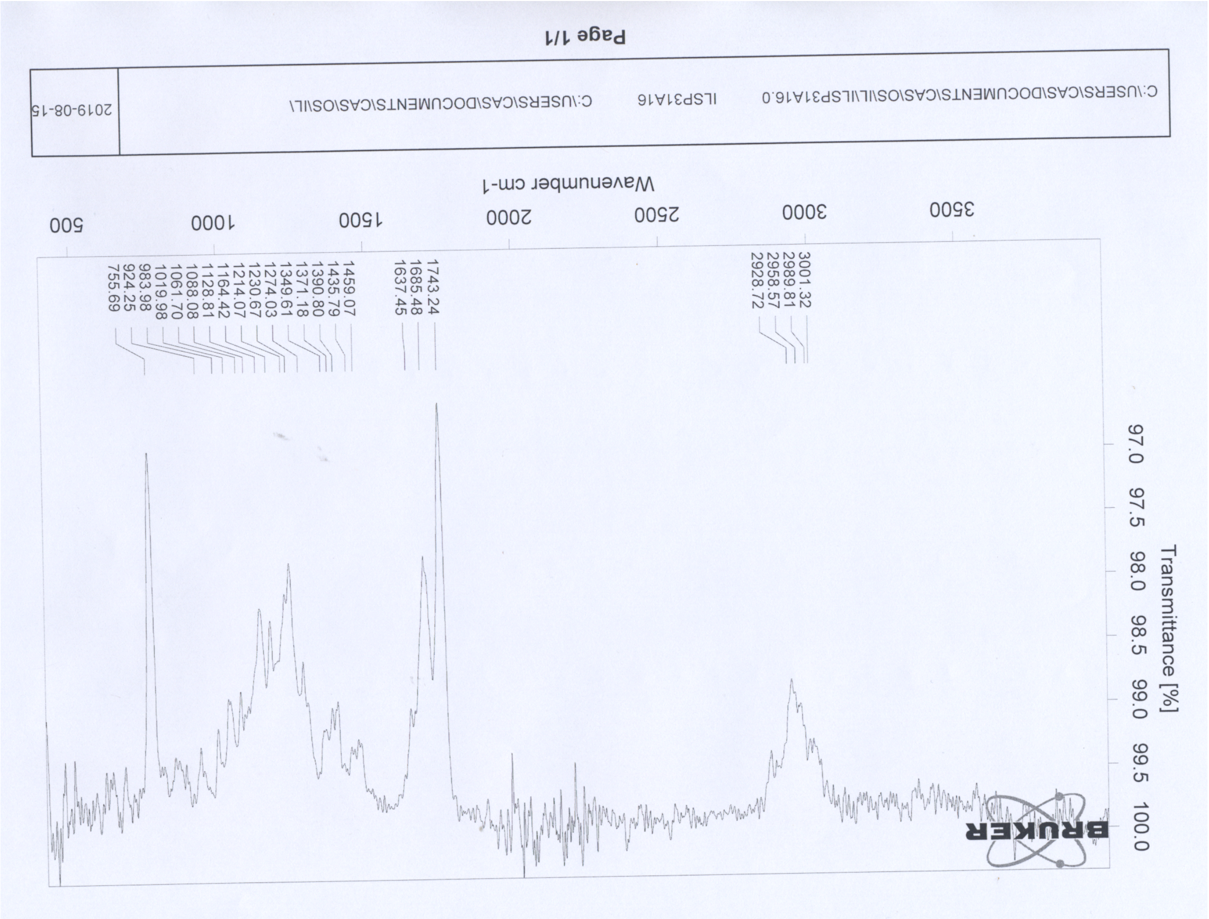

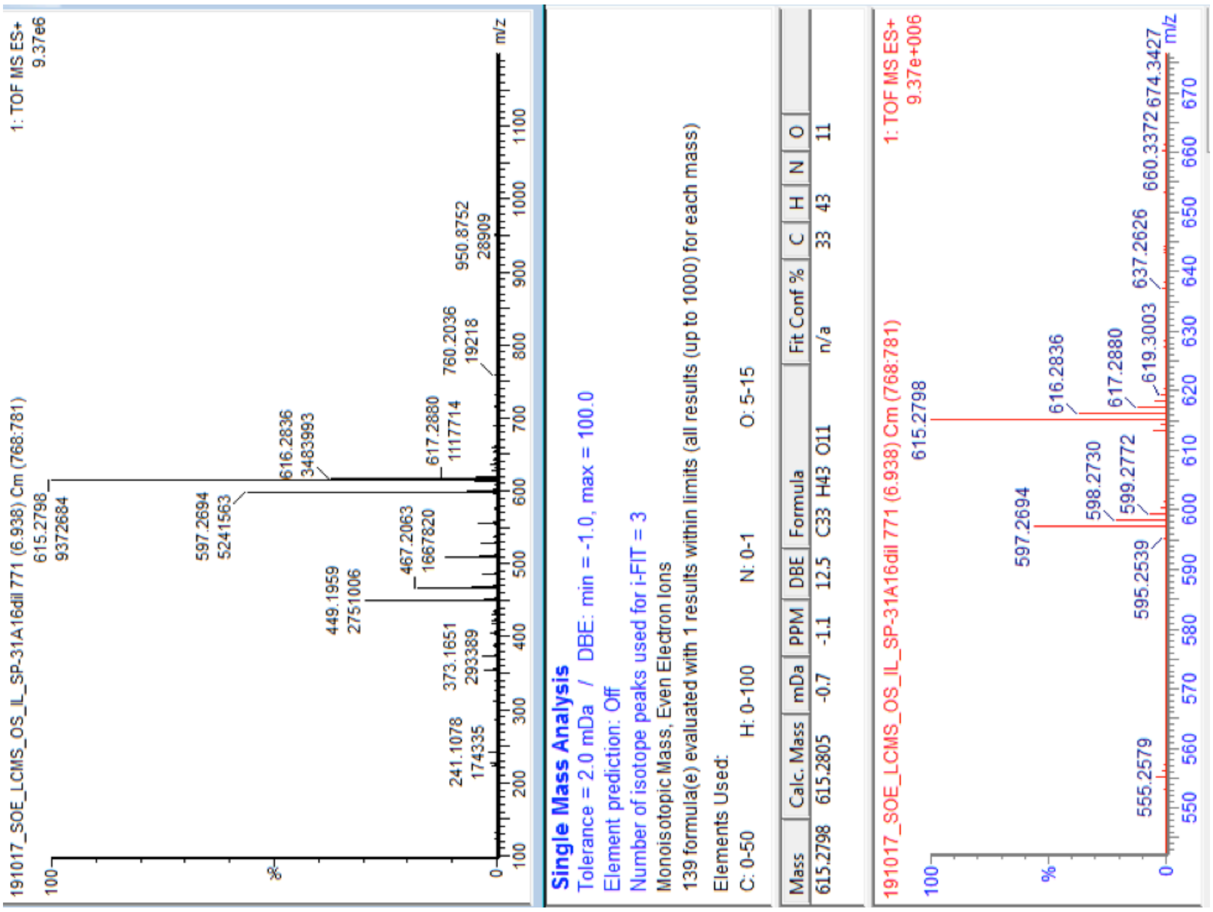

Supplement: Supplementary file 1 [file molecules-26-03070-s001.zip › molecules-1189833-supplementary.pdf]
